# Modelling the spatiotemporal complexity of interactions between pathogenic bacteria and a phage with a temperature-dependent life cycle switch

**DOI:** 10.1038/s41598-021-83773-1

**Published:** 2021-02-23

**Authors:** Halil I. Egilmez, Andrew Yu. Morozov, Edouard E. Galyov

**Affiliations:** 1 Hitit University, Faculty of Arts and Sciences, Department Of Mathematics, 19040 Çorum, Turkey; 2 University of Leicester, University Rd, Leicester, LE1 7RH UK; 3 Institute of Ecology and Evolution, Russian Academy of Sciences, 119071 Moscow, Russia

**Keywords:** Ecology, Evolution, Microbiology, Mathematics and computing, Applied mathematics, Computational science

## Abstract

We apply mathematical modelling to explore bacteria-phage interaction mediated by condition-dependent lysogeny, where the type of the phage infection cycle (lytic or lysogenic) is determined by the ambient temperature. In a natural environment, daily and seasonal variations of the temperature cause a frequent switch between the two infection scenarios, making the bacteria-phage interaction with condition-dependent lysogeny highly complex. As a case study, we explore the natural control of the pathogenic bacteria *Burkholderia pseudomallei* by its dominant phage. *B. pseudomallei* is the causative agent of melioidosis, which is among the most fatal diseases in Southeast Asia and across the world. We assess the spatial aspect of *B. pseudomallei*-phage interactions in soil, which has been so far overlooked in the literature, using the reaction-diffusion PDE-based framework with external forcing through daily and seasonal parameter variation. Through extensive computer simulations for realistic biological parameters, we obtain results suggesting that phages may regulate *B. pseudomallei* numbers across seasons in endemic areas, and that the abundance of highly pathogenic phage-free bacteria shows a clear annual cycle. The model predicts particularly dangerous soil layers characterised by high pathogen densities. Our findings can potentially help refine melioidosis prevention and monitoring practices.

## Introduction

Among major factors controlling bacterial numbers both in the wild and in artificial environments are natural enemies known as bacteriophages or phages. Phages are viruses that can specifically infect their host by attaching to particular bacterial receptors, injecting their genomic DNA (or RNA) into the host cell cytoplasm, and triggering a process that can lead to phage replication or integration of phage genome into the host chromosome. Viruses are considered to be the most abundant biological entity on our planet, and are known to be the key factor regulating the length and amplitude of algal and bacterial blooms in the ocean^1,2^. Targeting of undesirable bacteria using phages has been successfully used to overcome bacterial resistance to antibiotics in both food safety and medical applications^3,4^, and phages have be used to control the spread of deadly pathogens, such as *Vibrio cholerae* ^5^. However, the role of phages in regulating the number of bacteria in complex heterogeneous environments is still poorly understood. Mathematical modelling backed up by empirical data is a powerful tool for quantifying and forecasting the dynamics of bacterial hosts and their phages under changing environmental conditions^6^. In this paper, we use modelling to explore the regulation of the pathogenic bacterium, *Burkholderia pseudomallei*, by its dominant phage with a temperature-dependent life cycle switch in complex spatio-temporal environments.

The soil- and water-borne pathogen *B. pseudomallei* causes melioidosis, which is a serious human illness. It is a significant problem for the rice growing industry as the organism abounds in rice paddies, infecting agricultural workers, and killing around  45% of those infected. The overall number of deaths caused by the pathogen is estimated to be as high as 90,000 per year^7–10^. The US Centers for Disease Control and Prevention have identified *B. pseudomallei* as a potential biothreat agent^11^. *B. pseudomallei* is highly abundant in the natural environment and agricultural fields across the tropics, especially in Southeast Asia, particularly in Thailand and Laos, and northern Australia^7,12^. Unlike plankton blooms in the ocean, which are highly visible due to changes of the natural water colour, bacterial outbreaks in the soil are invisible. Due to the absence of coloration, these ‘invisible blooms’ can be easily overlooked by the general public and health control organisations. Until recently, the potential impact of phages on *B. pseudomallei* and its infectivity in the environment have been largely overlooked in the literature, but available empirical data suggest that phages can potentially control *B. pseudomallei* numbers in water or soil in a similar way to control of cyanobacterial blooms by marine viruses^13,14^. High phage numbers have been observed in soil containing *B. pseudomallei* in Thailand, Laos and Vietnam^14,15^, indicating that they possibly interact with and impact the bacteria.

**Figure 1:**
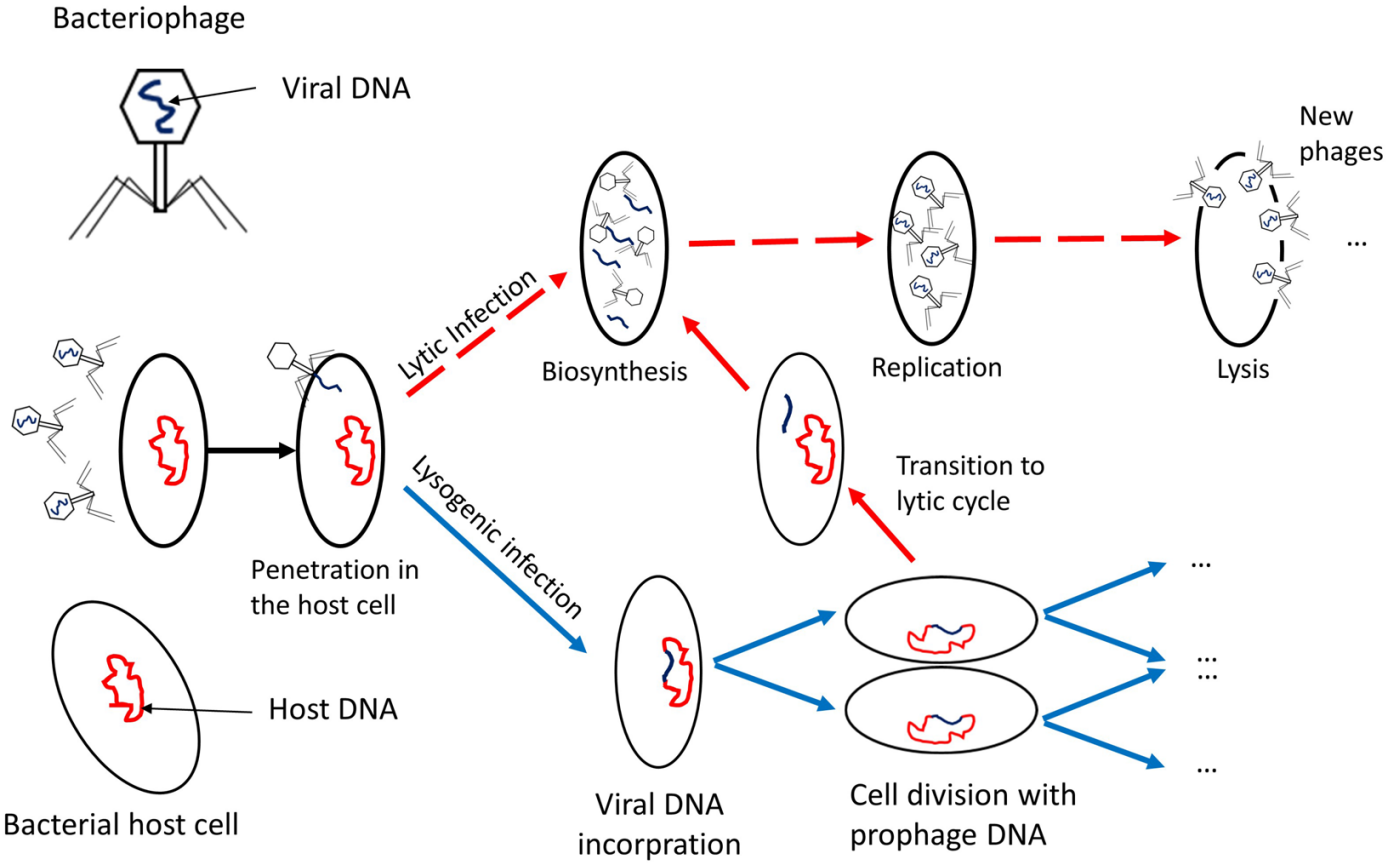
Schematic diagram explaining the two types of infection cycles of the phage (lytic and lysogenic) under the scenario of temperature-dependent lysogeny. At hot temperatures ($T>35$ °C), most phages follow a lytic cycle by killing the pathogen after the infection (shown by red dashed lines). At cooler temperatures ($T<35$ °C), infection by the phage mostly occurs via a lysogenic cycle where bacterial cells become phage-associated (shown by blue lines). However, an increase in the ambient temperature causes the bacteria-associated phages to enter the lytic state and lyse their hosts (shown by the red solid line).

Difficulties in understanding and predicting the efficiency of control of *B. pseudomallei* by its natural phages stem from the high complexity of the underlying biological system. In particular, *B. pseudomallei*-phage interaction is affected by the recently discovered ‘condition-dependent lysogeny’ that is characteristic for a clade of highly abundant phages^15^. Condition-dependent lysogeny is schematically depicted in Fig. 1. In ‘warm’ conditions the phages infect the pathogen and follow a lytic cycle (immediately killing the host cells), whereas at colder temperatures they lysogenise their bacterial hosts: in this case the phage remains in the cell without causing lysis^15^ and infected bacteria become phage-associated. Lysogenic (i.e. phage-associated) *B. pseudomallei* which enter a warm-blooded host would experience lysis of the pathogen, and would not be able to cause disease. In warm conditions most bacteria in the environment are in the ‘phage-free’ form and can cause disease when entering the human body. The switch between the lytic and the lysogenic infection cycles occurs at temperatures of around 35 °C^15,16^ which has important consequences for bacteria-phage interactions: daily and seasonal variation of the temperature in the main endemic areas of Southeast Asia and Australia should cause a transition between the two infection cycles. In this paper, we are interested in predicting natural control of *B. pseudomallei* by the phage by means of computational modelling in order to provide important knowledge to facilitate the natural bacterial control by the phage via adjusting the existing agricultural practices.

Mathematical and computational modelling of interactions between bacteria and their natural viruses has a long history starting from the seminal work of Campbell^17^ and later by Lenski et al.^18^. The amount of theoretical studies has increased dramatically within recent years (for a brief review see^6^), but surprisingly, systems involving temperature-dependent lysogeny have not received much attention until very recently. Egilmez et al. modelled *B. pseudomallei*-phage interactions under varying temperature using historic data from two endemic provinces in Thailand^16^. Their modelling study predicts that *B. pseudomallei* can exhibit annual blooms (outbreaks) during warm seasons, which positively correlates with the reported increased cases of melioidosis during these periods. The model, however, considered bacteria-phage dynamics in a well-mixed environment in the surface water of agricultural fields. As such, the model neglected the spatial aspect of the problem, while the fact that the key drivers of interactions, the temperature and the carrying capacity of bacteria in soil, have a pronounced spatial gradient in the vertical direction. Adding space might have a substantial effect on previous conclusions about disease prevention and monitoring practices from the well-mixed model.

Here we extend the model of temperature-dependent lysogeny developed by Egilmez et al.^16^ by adding a spatial dimension. We explore the spatiotemporal dynamics of bacteria-phage interactions in the upper layer of soil under daily and seasonal variation of the temperature. The dependence of model parameters on the temperature and the depth is taken from the available empirical literature and the temperature variation is taken from historical records for two endemic provinces of Thailand (Sa Kaeo and Nakhon). Using extensive computer simulations we explore spatiotemporal dynamics within a large range of biologically realistic model parameters. The spatial model is based on the classical reaction-diffusion framework (PDEs), and despite the overall complexity of the system, the model can be still considered to be parsimonious compared to highly complex soil models. The reaction-diffusion approach can be applied since the considered non-homogeneous system is under a strong external forcing (by means of daily and seasonal temperature variation).

Our model predicts that the system undergoes clear seasonal cycling behaviour with outbreaks of phage-free pathogenic bacteria occurring at the onset of the hot season in both provinces considered. We find that the vertical profiles of phage-free and infected bacterial cells (in both lysogenic and lytic states) show a non-monotonous pattern: bacterial blooms with a high density of phage-free *B. pseudomallei* are observed at some intermediate depths, which can have important consequences for the safety of agricultural workers. Interestingly, enrichment of the environment in the model (e.g. by using agricultural fertilisers) can cause irregular spikes in phage-free bacteria numbers, signifying a higher risk of disease. This theoretical study re-enforces the early hypothesis that temperature-responsive phages could play a key role in regulating bacterial numbers both daily and across seasons^16^. It provides possible ways to improve the existing monitoring of *B. pseudomallei* in soil using information on the vertical distribution of the pathogen.

## Methods

### Model equations

We introduce a spatiotemporal model to describe the bacteria-phage interaction in the upper part of the soil with the depth *H* (we consider $H=1$ m) in a typical agricultural field. Here we consider a 1D model where all abiotic and biotic components depend on time *t* and vertical coordinate *h*. The biotic component of the model consists of 4 compartments: phage-free bacteria (*S*) susceptible to infection by the phage, bacteria infected by the phage in its lysogenic ($I_1$) and lytic ($I_2$) states, and free phages (*P*). The total density of the host bacterial populations *N* is defined as $N = S + I_1 + I_2$. The schematic diagram illustrating bacteria-phage interactions is similar to that of Egilmez and co-authors^16^. The local species interactions are described based on the classical modelling approach^6,19^. Our spatiotemporal model is of reaction-diffusion type and is described by the following equations

1 $$\begin{aligned} \begin{aligned} \frac{\partial S(t,h)}{\partial t}&= D_b \frac{\partial ^2 S(t,h)}{\partial h^2} +\alpha (T) S(t,h) \Big [1-\frac{N(t,h)}{C(h)}\Big ] - K_S S(t,h)P(t,h), \\ \frac{\partial I_1(t,h)}{\partial t}&= D_b \frac{\partial ^2 I_1(t,h)}{\partial h^2} + {\overline{\alpha }}(T) I_1(t,h) \Big [1- \frac{N(t,h)}{C(h)}\Big ] + K_1(T) S(t,h) P(t,h) - \lambda _1(T) I_1(t,h), \\ \frac{\partial I_2(t,h)}{\partial t}&= D_b \frac{\partial ^2 I_2(t,h)}{\partial h^2} + K_2(T) S(t,h) P(t,h) + \lambda _1(T) I_1(t,h) - \lambda _2 I_2(t,h), \\ \frac{\partial P(t,h)}{\partial t}&= D_P \frac{\partial ^2 P(t,h)}{\partial h^2} -K N(t,h) P(t,h) - \mu P(t,h) + b \lambda _2 I_2(t,h). \end{aligned} \end{aligned}$$

In the above model, we parameterise the growth of susceptible bacteria via a standard logistic growth function^6^, where $\alpha$ is the maximal per capita growth rate and *C* is the carrying capacity of the environment; we assume that *C*(*h*) varies with depth. Infection of *S* by phages *P* at low temperatures results in lysogeny which is described by a mass action term $K_s S(t,h) P(t,h)$. The growth of lysogenic bacteria $I_1$ is described by a logistic function as in the case of *S*; however, with a different maximal growth rate ${\overline{\alpha }} (T)$ as detailed in the next subsection. At high temperatures, the transition from the lysogenic to the lytic cycle of infection occurs: this is described by the term $\lambda _1 (T) I_1(t,h)$. Infection by the phage via the lytic cycle is modelled by the term $K_2 (T)S(t)P(t)$. The death rate of infected bacteria due to lysis is modelled by $\lambda _2 (T) I_2$. The lysis of a bacterium results in the release of *b* new phages, the the burst size^6^. In the equation for *P*, *KN*(*t*)*P*(*t*) stands for the loss of phage due to binding to any type of bacteria (for simplicity, we assume that there is no saturation in binding). Finally, $\mu P(t,h)$ is the natural mortality or deactivation of phages.

According to this framework, the vertical displacement of the phage and bacteria are modelled by a diffusion term (first term in each equation), where $D_b$ and $D_P$ are the diffusion coefficients of bacteria and phage, respectively. The variation of the temperature *T* across the soil is described by the heat equation

2 $$\begin{aligned} \frac{\partial T(t,h)}{\partial t} = D_h \frac{\partial ^2 T(t,h)}{\partial h^2}, \end{aligned}$$

where $D_h$ is the diffusion coefficient of heat transfer (see more detail in the next section). Models (1)–(2) should be supplied with appropriate boundary conditions. We assume that the model has the zero-flux boundary condition for all biotic components (bacteria and phage) at $h=0$ and $h=H$. For the temperature, we consider Dirichlet boundary conditions such that $T(t,0)= T_s (t)$ and $T(t,H)= T_H$, where $T_s (t)$ is the surface temperature and $T_H$ is a constant temperature in deeper soil layers.

### Parameterisation of equation terms

Next we describe the functional forms of the dependence of model parameters on the temperature and the depth. Following the previous study^16^, we assume that the maximal bacterial growth rates $\alpha (T)$ and ${\overline{\alpha }}(T)$ are described by

3 $$\begin{aligned} \alpha (T)= & {} \exp \left (-\frac{(T-T_0)^2}{2\sigma ^2}\right )\alpha _{\text {max}}, \end{aligned}$$

4 $$\begin{aligned} {\overline{\alpha }}(T)= & {} \alpha (T) \left [1-\frac{T^n}{T_1^n + T^n}\right ] = \alpha _{\text {max}}\exp {\left (-\frac{(T-T_0)^2}{2\sigma ^2}\right )} \left [1-\frac{T^n}{T_1^n + T^n}\right ], \end{aligned}$$

where $T_0=38.2 \ ^\circ \text {C}$ is the optimal temperature; $T_1=34.8 \ ^\circ \text {C}$ is the temperature corresponding to the switch between the lytic and the lysogenic cycles; $\alpha _{\text{max}}=23 \ \text {day}^{-1}$ is the maximal possible growth, $\sigma =9.1 \ ^\circ \text {C}$ describes the decay of growth with temperature *T*^16,20^.

In the equation for ${\overline{\alpha }}(T)$, we assume that at a high temperature normal cell division of $I_1$ stops since there is a transition to a lytic state in bacteria. In the soil bacteria grow anaerobically or microaerophillically, and the growth rates of *B. pseudomallei* under such conditions are yet to be studied. For simplicity they are assumed to be the same as under aerobic conditions. Realistic values of the above parameters are listed in Table 1. Note that in the model both $\alpha (T)$ and ${\overline{\alpha }}(T)$ are in fact effective growth rates of the bacterial populations, i.e. they incorporate the replication of cells and as well as their mortality.

**Table 1:** Parameters used in the model along with their units and ranges.

Symbol	Meaning	Unit	Range	Default Value
$D_b$	Bacteria diffusion coefficient in soil	$\text {cm}^2 \ \text {day}^{-1}$	$10^{-12}{-}10^{-3}$	$10^{-7}$
$D_P$	Phages diffusion coefficient in soil	$\text {cm}^2 \ \text {day}^{-1}$	$10^{-12}{-} 10^{-3}$	$10^{-5}$
$D_h$	Heat diffusion coefficient in soil	$\text {cm}^2 \ \text {day}^{-1}$	$-$	67
$\rho _\text {s}$	Bulk density	$\text {kg}/\text {m}^3$	$-$	$1.1 \times 10^{3}$
$C_\text {ps}$	Specific heat	J/kg K	$-$	$1.1 \times 10^{3}$
$k_\text {s}$	Thermal conductivity in soil	W/m K	−	0.1
$\alpha _{\text {max}}$	Maximum growth rate of bacteria	$\text {day}^{-1}$	$19{-}27$	23
$C_\text {surf}$	Bacteria carrying capacity near the surface	$\text{cell}/ \text {ml}$	$1 \times 10^{6}{-} 1 \times 10^{7}$	$1 \times 10^{6}$
$C_{0}$	Bacteria carrying capacity at large depths	$\text{cell}/ \text {ml}$	$1 \times 10^{4}{-}1 \times 10^{7}$	$1 \times 10^{6}$
$\sqrt{B}$	Inverse characteristic length of *C*(*h*)	$\text {cm}^{-2}$	−	$7.5 \times 10^{-4}$
*K*	Phage adsorption rate	$\text {ml}^{-1} \text {day}^{-1}$	$5 \times 10^{-8}{-}5 \times 10^{-7}$	$1 \times 10^{-7}$
$K_S$	Effective per bacteria contact rate	$\text {ml}^{-1} \text {day}^{-1}$	−	$\epsilon \times 10^{-7}$
$\epsilon$	Adsorption efficiency	−	−	0.3
${\lambda _1}_{\text {max}}$	Maximum lysogenic process rate	$\text {day}^{-1}$	$19.1{-}27.2$	23
$\lambda _2$	Constant lysis rate	$\text {day}^{-1}$	−	20
*b*	Phage burst size	−	$50{-}200$	105
$T_0$	Optimum temperature for growth and lysis	$^\circ \text {C}$	$35.6{-}50.6$	38.2
$T_1$	Optimum transition temperature	$^\circ \text {C}$	$34.81{-} 34.84$	34.8
$\sigma$	Standard deviation of growth rate	$^\circ \text {C}$	$6.7{-}17.4$	9.1
$\mu$	Mortality rate of phages	$\text {day}^{-1}$	$0.1{-}15$	3
*n*	Transition width	−	$53.7{-}56.3$	55

**Figure 2:**
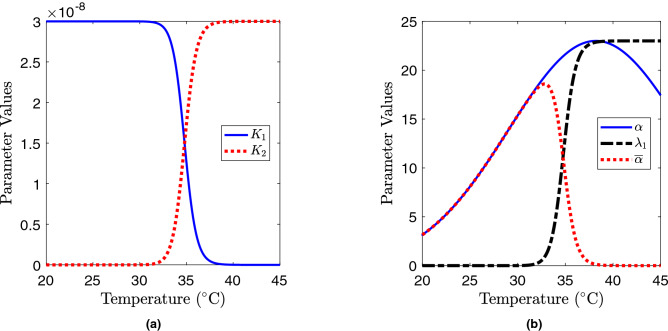
(**a**) Temperature dependence of the adsorption constants $K_i$ ($i=1,2$) of the phage (measured in $\text {ml}^{-1} \text {day}^{-1}$). (**b**) Growth rates of susceptible $\alpha (T)$ and lysogenic ${\overline{\alpha }}(T)$ bacteria and the transition rate $\lambda _1(T)$ from the lysogenic cycle to the lytic cycle (measured in $\text {day}^{-1}$). The corresponding analytical expressions for the temperature dependence are given by (3)–(5).

The overall adsorption rate of the phage *K* is estimated as $2 \times 10^{-7} \ \text {ml}^{-1} \text { day}^{-1}$ from Egilmez et al.^16^. The adsorption constants $K_1 (T)$, $K_2 (T)$ and the transition rate from lysogenic to lytic cycle $\lambda _1(T)$ depend on temperature as follows^16^:

5 $$\begin{aligned} K_1(T)&= \left(1-\frac{T^n}{T_1^n + T^n}\right) K_S, \nonumber \\ K_2(T)&= \frac{T^n}{T_1^n + T^n} K_S, \nonumber \\ \lambda _1(T)&= \frac{T^n}{T_1^n + T^n} {\lambda _1}_{\text {max}} , \end{aligned}$$

where $K_S$ is the maximal phage adsorption constant ($K_S=\epsilon K$ where $\epsilon =0.3$ is the adsorption efficiency) and $\lambda _{1_\text {max}}=23 \ \text {day}^{-1}$ is the maximal transition rate which is assumed to be equal to the maximal growth rate of the bacteria^16^. The lysis rate of bacteria $\lambda _2=20 \ \text {day}^{-1}$ (depending on 50 min latency time^13^) and the burst size $b = 100$ in the model are assumed to be constant^16^. The temperature dependence of $\alpha (T)$, ${\overline{\alpha }}(T)$, $K_1 (T)$, $K_2 (T)$ and $\lambda _1(T)$ are shown in Fig. 2. The mortality rate of phages $\mu$ is high near the surface due to ultraviolet radiation, but the role of ultraviolet radiation becomes negligible starting from a depth of a few centimetres because sunlight cannot penetrate the soil. For the above reason, we can assume $\mu =3 \ \text {day}^{-1}$ to be constant.

The carrying capacity *C* of the bacteria varies with the depth of the soil, according to empirical observations^21–23^. This can be explained by the fact that the humus, oxygen, nitrogen contents, or/and water content in the soil generally decrease with depth^24^. We use a combined approach to parameterise *C*(*h*) based on the available empirical data. We assume that in the absence of phages, the bacteria achieve numbers close to the carrying capacity at a given depth. Firstly, we parameterise the dependence of the overall bacterial load on depth in paddy soils in Southern Asia using the existing data^22^. Then we re-scale the obtained curve based on the available observations of *B. pseudomallei* at a depth $h=30 \ \text {cm}$^25,26^. We approximate *C*(*h*) using the following simple Gaussian-type curve

6 $$\begin{aligned} C(h)=(C_\text {surf} -C_0)\exp (-B h^2)+C_0, \end{aligned}$$

where $C_\text {surf}$ gives the maximal number of bacteria near the surface (*h*), *B* determines how fast the bacterial abundance decreases with depth, $C_0$ is background bacterial density which takes into account the fact that bacteria can survive even at large depths (e.g. $h=100 \ \text {cm}$). Based on our estimates (see supplementary material SM1 for more detail), we will use the following parameter values as defaults: $C_\text {surf} = 1 \times 10^6$ $\text {cell/ml}$, $B=7.5 \times 10^{-4}$ $1/{\text{cm}}^2$, $C_0=10^4$ $\text {cell/ml}$ . One can easily see that *C*(*h*) has a maximum at the surface and monotonically decreases with depth. We assume that the carrying capacity of the environment is not influenced by seasonal variation.

The coefficient $D_h$ in the equation for the temperature distribution can be estimated as follows. Generally, $D_h$ is related to $\rho _s$, $C_{\rho s}$ and $k_s$ which are the bulk density, specific heat and thermal conductivity in soil, respectively, i.e. $D_h=k_s/(\rho _s C_{\rho s)}$. We use the estimates for $\rho _s$, $C_{\rho s}$ and $k_s$ from^27^ which gives $\rho _s=110.52 \ \ \text {kg}/\text{m}^3$, $C_{\rho s} = 1130$ $\text {J/kg K}$ and $k_s = 0.0967$ $\text {W/m K}$ and, for the diffusion coefficient $D_h=7.7 \times 10^{-8}$ $\text {m}^2 \text{s}^{-1}$. The variation of $T_s$—the surface temperature—is obtained from the historical weather report for the surface^16^. The bottom boundary temperature $T_H$ at $h=H=1 \ \text {m}$ is considered to be $22 \ ^\circ \text{C}$. The initial value of the temperature distribution $T_s (0)$ is assumed to be linear, but this assumption does not affect long-term temperature dynamics.

The paddy fields in which we model the bacteria-phage interactions are flooded lands, where the soil is either mud or muddy water. Many factors can affect vertical dispersal of bacteria and phages in such soil. For instance, rain water can carry bacteria and phage up or down in the soil, which can be mathematically modelled by adding an advection term; however, for simplicity we ignore such effects in this paper. We also assume the phage and bacteria vertical diffusion coefficients to be constant; however, it is rather hard to provide accurate estimates for $D_p$ and $D_b$. In water, the diffusion coefficient of bacteria and phages can be estimated as $3.6\times 10 ^{-10} \ \text {m}^2 \text{s}^{-1}= 0.3 \ \text {cm}^2 \text{day}^{-1}$ and $2.8 \times 10^{-12} \ \text {m}^2 \text{s}^{-1}= 0.002 \ \text {cm}^2 \text{day}^{-1}$, respectively^28^, but the diffusivity in soil should be smaller than this. As such, these values should be considered as upper limits for $D_P$ and $D_b$, with the actual coefficients being orders of magnitude smaller. We undertook simulations with different combinations of diffusion coefficients in this range, and found that the patterns of vertical distribution do not largely depend on the diffusion coefficients provided $D_P< 10^{-3} \ \text {cm}^2 \text{day}^{-1}$ and $D_b < 10^{-2} \ \text {cm}^2 \text{day}^{-1}$, due to the strong external forcing of the system by temperature (see “Results” section for details).

In our numerical simulations, we use both explicit and implicit numerical schemes. We take a 0.1 cm spatial step size to get a proper resolution. We separately compute the heat equation to define *T*(*t*) with a smaller time resolution and then apply the temperature obtained to model bacteria-phage interactions for a larger time resolution (for example, $\Delta t \cong 7 \times 10^{-5}$ day). We compute the average densities of the species (both in terms of spatial and temporal averaging) using a numerical right Riemann sum. The accuracy of our numerical simulation was verified by reducing both time and space steps and comparing the results obtained. We use daily and seasonal variation of temperatures (for the period of 2013–2016) in the provinces of Nakhon Phanom and Sa Kaeo in Thailand to parameterise the model (http://www.worldweatheronline.com). The unit of the densities of bacteria and phages are cells/ml. The summary of model parameters as well their values are provided in Table 1.

## Results

### Modelling temperature variation in soil

**Figure 3:**
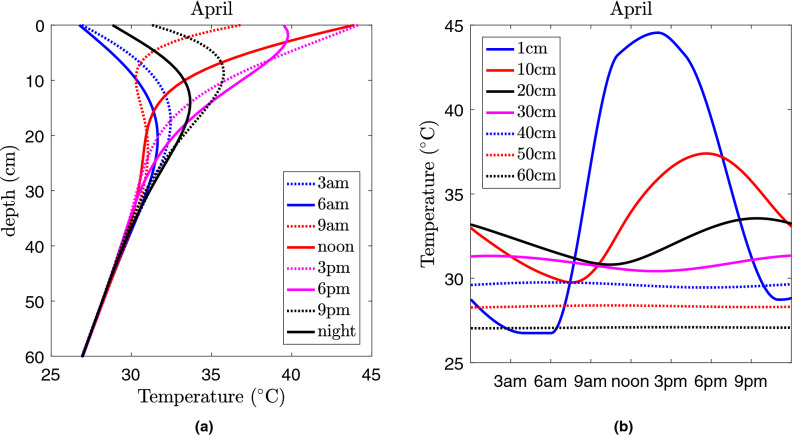
Vertical distribution of the temperature across the soil (**a**) and daily temperature variation at a fixed depth of the soil (**b**) for the first day of April in a typical field in Nakhon Phanom province in Thailand predicted by the heat equation (2) using historical surface temperature data for the period of 2013–2016.

Using Eq. (2) and historical data of temperature variation near the soil surface during the 3 year period considered, we explored the daily spatiotemporal variation of *T*(*h*, *t*) as well as dynamics across seasons. Figure 3a and 2S in SM2 show examples of the vertical temperature distribution at different times of the day for the first day of January, April, July and October in the Nakhon Phanom province in Thailand. In the other province Sa Kaeo, the temperature variation exhibits a similar spatiotemporal pattern which is not shown here for brevity. It is apparent that the temperature exhibits daily oscillations until depths of around $h=40 \ \text {cm}$. At greater soil depths the temperature gradually decreases towards the boundary value of $T_H=22$ °C uniformly across seasons.

Temperature variations observed at several fixed depths are shown in Fig. 3b and 3S in SM2, plotted for the same days as those presented in Fig. 3a and 2S in SM2. We compared the range of temperature variation predicted by the model with reported field data in Thailand^29^, and found a good overall agreement between theoretical and empirical values which allows us to substitute the theoretical values of *T*(*h*, *t*) into model equations (1). The main conclusion from Figs. 2, 3 is that variation of temperatures both in time and space occurs around the critical value of $T_1$, which describes the switch between the lytic and the lysogenic infection cycles in the highly abundant phage of *B. pseudomallei*. One can see that the temperature-dependent switch occurs in the range of depths from $h=0$ to $h=20\; \text{cm}$. This fact has profound consequences for bacteria-phage interaction, which are discussed below.

### Seasonal dynamics of bacterial and phage numbers

**Figure 4:**
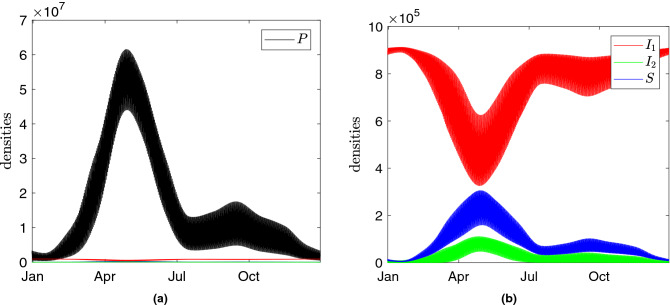
(**a**,**b**) Daily and seasonal temporal dynamics of bacteria and phage numbers within the upper 20 cm of the soil predicted by the model for Nakhon Phanom province in Thailand. Model parameters are taken from Table 1 as default values. The unit of the densities of bacteria and phages are cell/ml and phage/ml, respectively.

Using the above temperature variation patterns, we run simulations of the model to obtain predicted bacterial and phage densities. The dynamics of all components for the default parameter values in Table 1 are shown in Fig. 4, plotted for Nakhon Phanom province in Thailand. In this figure, we present the temporal dynamics of the phage and bacteria densities which are spatially averaged from the surface to the depth $h=20$ cm (note that the considered upper soil layer is characterised by the most pronounced daily oscillations in temperature, see Figs. 3 and SM2). One can see that the overall population of susceptible bacteria *S* in the upper soil layers demonstrates a pronounced seasonal trend with a peak in May–June. This can be explained by the fact that higher temperatures promoting bacterial growth are observed during this time. The overall amount of free phages in the soil follows the annual pattern of susceptible bacteria: the presence of a large number of *S* at high temperatures results in a massive replication of *P* across the soil layers. The lysogenic bacteria $I_1$ show the opposite dynamics to both susceptible bacteria *S* and bacteria in the lytic state $I_2$ by following the temperature variation controlling the switch between the two infection cycles.

**Figure 5:**
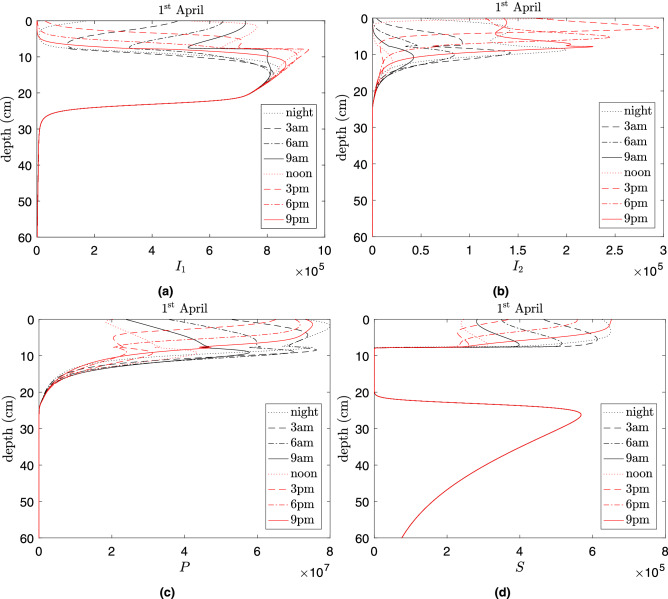
Vertical distribution of infected bacteria predicted by the model (**a**): in lytic $I_1$, (**b**): in lysogenic $I_2$, (**c**): phage *P* and (**d**): susceptible bacteria *S* in the soil across the day of April 1st predicted by the model calculated for Nakhon Phanom province. Model parameters are taken from Table 1 as default values. Note that the curves in (d) overlap for depths *h*>20cm. The unit of the densities of bacteria and phages are cell/ml and phage/ml, respectively.

Figure 5 shows the vertical profiles of the densities of all types of bacteria and the phage on April 1st (the profiles of densities of microorganisms for the other seasons of the year are shown in supplementary material SM4). The profile *S*(*h*) is characterised by two pronounced maxima: a narrow peak in the upper soil layers, and a lower one at a depth of approximately $h=30 \ \text {cm}$. Lysogenic bacteria $I_1$ are dominant in the range of depths from a few centimetres in the upper surface layers to the depth where *S* achieves its deep maximum. The reason that *S* can outcompete $I_1$ at deeper soil layers—despite the fact that they have the same growth rate—is because of the lysis of $I_1$ which occurs during warm periods. Bacteria in the lytic state $I_2$ are located mostly near the surface which coincides with higher phage densities. The vertical profile of the free phage can also have several peaks as well, but these are mostly located near the surface within depths up to approximately $h=15 \ \text {cm}$: at deeper depths the phage can only persist via a lysogenic mode (inside bacteria).

Daily temperature variation causes high amplitude oscillations of susceptible bacteria *S* near the surface with the lowest and the highest abundances being at mid-day and late evening, respectively. These oscillations are strongly correlated with those of the phage density *P*. The overall pattern of the vertical distribution of microorganisms remains similar across seasons with only minor alterations. For example, in April–May the locations characterised by a high density of *S* extend from the surface to deeper depths. This results in peaks of the overall amount of pathogenic bacteria shown in Fig. 4. Interestingly, the lower peak of *S* remains constant throughout all seasons. Our simulations demonstrate a similar pattern of daily and annual dynamics of bacterial and phage numbers for Sa Kaeo province (see supplementary material SM6).

### Dependence of system dynamics on model parameters

**Figure 6:**
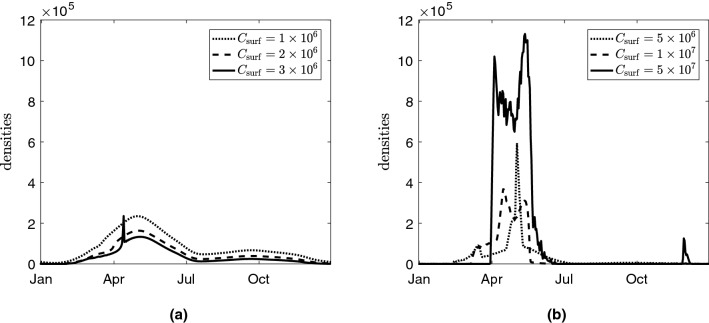
Daily average densities of susceptible bacteria *S* within the upper 20 cm of the soil calculated for different values of carrying capacity $C_\text {surf}$ (Nakhon Phanom province): The corresponding values of $C_\text {surf}$ (measured in cell/ml) are provided in figures: (**a**) $C_\text {surf}= 1 \times 10^{6} \;\text{cell}/\text {ml}$, $C_\text {surf}= 2 \times 10^{6} \;\text{cell}/\text {ml}$, $C_\text {surf}= 3 \times 10^{6}\; \text{cell}/\text {ml}$. (**b**) highly enriched environment, $C_\text {surf}= 5 \times 10^{6} \;\text{cell}/\text {ml}$, $C_\text {surf}= 1 \times 10^{7} \; \text{cell}/\text {ml}$, $C_\text {surf}= 5 \times 10^{7}\; \text{cell}/\text {ml}$. The unit of the density of *S* is cell/ml.

We varied key model parameters to assess their influence on system dynamics. Increasing the carrying capacity of bacteria $C_\text {surf}$ (e.g. due to excessive use of fertiliser in agricultural fields) results in the appearance of sharp peaks of the abundance of *S* during warm seasons due to the higher temperatures in the top soil. In this case, most lysogenic bacteria switch to the lytic cycle and then die. Figure 6 presents the annual dynamics of the bacterial density in the top 20 cm of the soil for increasing values of the carrying capacity. Unlike Fig. 4, the densities are averaged through the entire day. The observed non-smoothness of the curves for high values of $C_\text {surf}$ occurs due to high irregularity in the daily oscillations of *S*, $I_1$, $I_2$, and *P*. Enrichment of the environment by adding nutrients generally promotes non-periodic daily oscillations and sudden bursts of bacteria. It also increases the amplitude of peaks of susceptible pathogenic bacteria (not shown here for brevity). This mechanism is similar to the classical paradox of enrichment in predator-prey systems^30–33^. High amplitude oscillations of *S* would signify a higher risk of disease acquisition.

**Figure 7:**
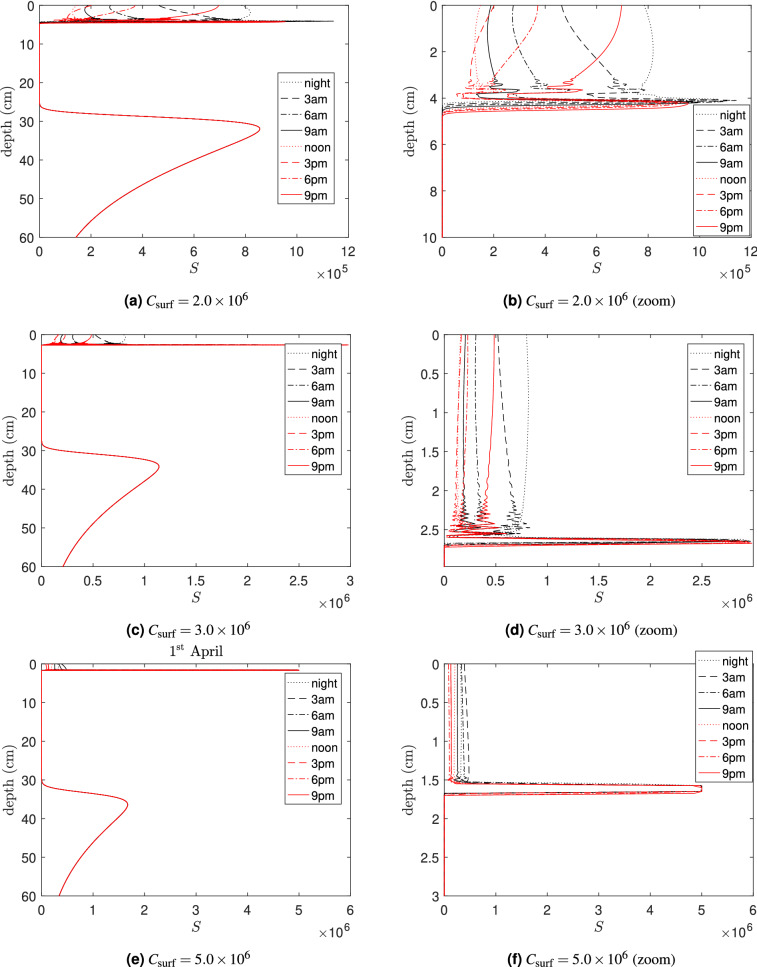
Influence of the carrying capacity on the vertical distribution of susceptible bacteria *S* in the soil predicted by the model calculated for Nakhon Phanom province. The left panel shows vertical distributions in the top 60 cm whereas the right panel presents zooms of the same profiles near the surface. The corresponding values of $C_\text {surf}$ (measured in cell/ml) are provided in figures. The graphs show the spatial distributions predicted for April 1st. The unit of the density of *S* is cell/ml.

Enrichment of the soil largely alters the vertical distribution of bacteria and phages shown in Fig. 7 (for the vertical profiles of other components see SM3). The range of depths with high densities of *S* near the surface shrinks with an increase of $C_\text {surf}$. Moreover, the profiles show a narrow and sharp peak of *S* which presents potential danger for agricultural workers (note that such peaks occur at all times). Analysis of the model for a fixed temperature shows that the existence of these sharp peaks is related to a Hopf bifurcation which occurs when the carrying capacity is varied.

Other important parameters of the model are the diffusion coefficients of microorganisms in the soil. We find that, surprisingly, variation of $D_p$ and $D_b$ within the ranges $10^{-11}< D_P < 10^{-3} \ \text {cm}^2 \text{day}^{-1}$ and $10^{-10}< D_b < 10^{-2} \ \text {cm}^2 \text{day}^{-1}$ results in only slight changes in the dynamics (the corresponding graphs are presented in supplementary material SM5). Only in cases where the diffusion coefficients become substantially large $(D_{b,p} > 10^{-2} \ \text {cm}^2 \ \text{day}^{-1})$ does the vertical profile of *S* become altered. This can be explained by a strong external forcing on the system by the temperature variation: the system essentially becomes a set of independent oscillators synchronised by external forcing (i.e. local interactions between phages and bacteria involving oscillatory dynamics). This holds true for any ratio of $D_p/D_b$ provided the coefficients are small in the absolute value. This finding strengthens our theoretical predictions, since accurate estimates for values of $D_p$ and $D_b$ are not available in the literature.

**Figure 8:**
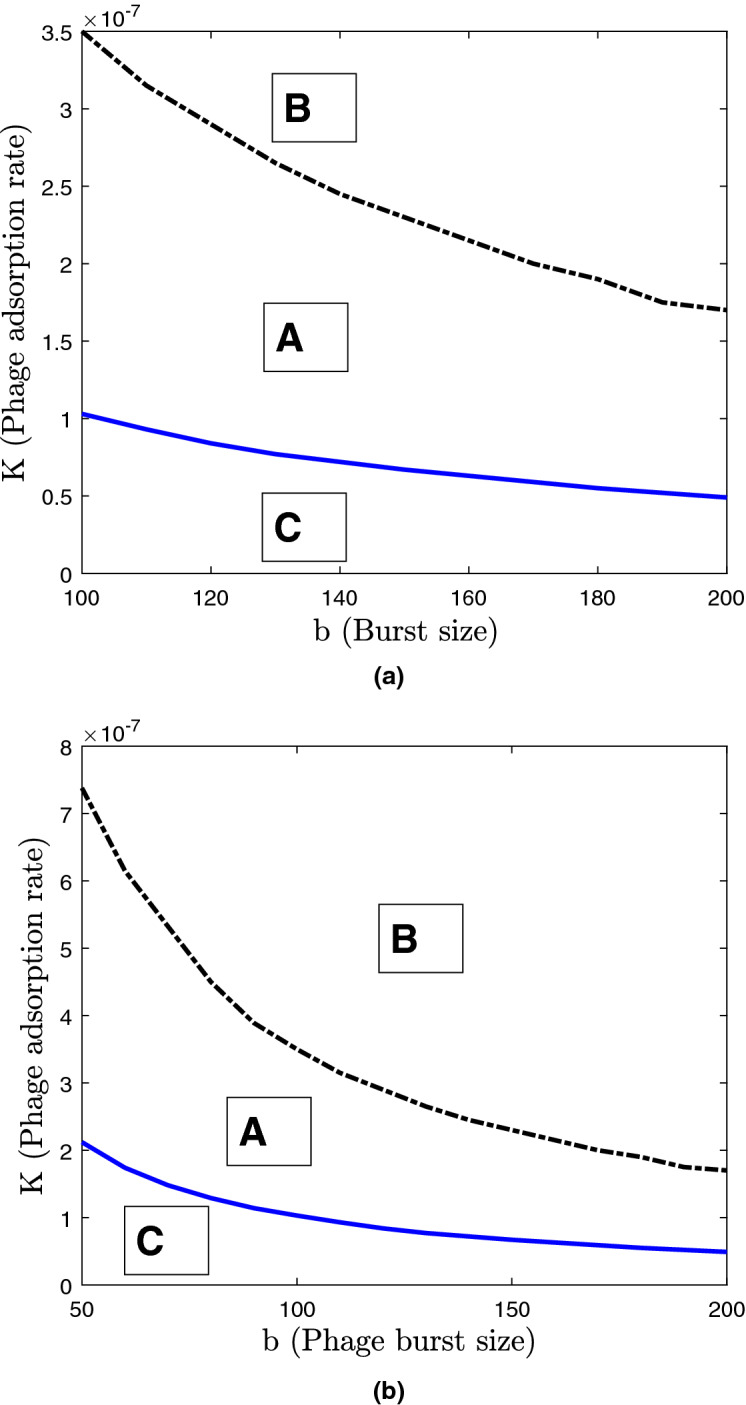
Bifurcation diagrams showing dynamical regimes in the model (Nakhon Phanom province) depending on the parameters $\mu$ (the morality rate of phages)-$C_\text {surf}$ (carrying capacity on the surface) and *K* (overall phage adsorption rate) and *b* (phage burst size). The classification of regimes A–C is the following. Regime “A” corresponds to periodic daily variations of species densities; for regime “B” species shows irregular oscillations; regime “C” signifies the extinction of phages. Other model parameters are set at default values.

Finally, we investigated the role of three other key model parameters describing the efficiency of phages: the phage mortality $\mu$, the burst size *b* and the adsorption constant *K*. The results are summarised in the two following diagrams shown in Fig. 8. The other parameters are kept constant to their default values. In the diagrams we categorised the pattern of dynamics into three different dynamical regimes. Regime A corresponds to dynamics exhibiting oscillations in species densities due to daily and seasonal variations, as seen in Figs. 4, 5. Regime B corresponds to the pattern of dynamics shown in Fig. 7. In this case, fluctuations in species densities do not match daily and/or seasonal variations but are highly irregular. These two regimes can be distinguished using the dynamical patterns of daily average densities. Finally, regime C corresponds to the extinction of the free phage in the system. This occurs because the average phage replication is lower than its mortality. From the diagrams one can conclude that the phage goes extinct for large values of mortality, small adsorption rates, and low carrying capacities in the system. For a fixed nutrient level (a constant $C_\text {surf}$), low mortality rates of phages result in irregular dynamics with high spikes in bacterial densities.

## Discussion and conclusions

In this paper we make a theoretical exploration of the natural control of bacteria by their phages under the recently discovered scenario of temperature-dependent lysogeny^15^ in a heterogeneous environment such as soil. The practical importance of the problem comes from the fact that melioidosis—the disease caused by infection of the pathogenic bacteria *Burkholderia pseudomallei*—is now estimated to be the third most fatal in Southeast Asian countries^7,12^. Despite available empirical data indicating high abundance of phages in the soil of endemic areas in Thailand (and in Southeast Asia overall), the natural control of the pathogen by phages has been largely overlooked in the literature so far and this study is intended to partly bridge the existing gap. Note that more complex mathematical models exist, describing microbial transport in the soil as a series of attachment and detachment processes or complicated random walks^34,35^. Other approaches include integro-differential equations describing the spatial effects of replication delay of the phage^36^. Here, however we address the problem using a parsimonious model based on the reaction-diffusion approach which is well-known in mathematical ecology^37^. We argue that implementation of this approach is justified because of the low sensitivity of the model prediction to variation in the diffusion coefficients (SM5), with strong external forcing and local interactions being the main drivers of the system dynamics. In particular, the influence of the external forcing of temperature on the system becomes facilitated by the temperature-dependent switch between the two infection cycles, which provides an extra degree of predictability for microbial dynamics: in the system with a single type infection cycle (lytic or lysogenic), variation of the temperature would only affect the growth rates of bacteria.

We find that generally the densities of bacteria and phage in soil show a seasonal trend both in terms of their vertical distribution and the total numbers. In particular, the model predicts that during the warmest period of the year (April–July), the densities of phage-free bacteria and phages increase while the density of lysogenic bacteria decreases. This would signify a higher risk of infection during warm periods of the year since phage-free bacteria can readily infect warm-blooded hosts, while lysogenised bacteria are likely to be killed by the induced phage upon entry. The spatial model overall confirms the seasonal pattern reported earlier based on the non-spatial model^16^. However, the spatial model shows more pronounced seasonal peaks of pathogenic bacteria than the non-spatial one (this follows from comparison of patterns with the previously published non-spatial model^16^). Note that in the non-spatial model—considering interactions in the surface water of agricultural fields—the strength and variation of solar UV radiation was found to be a major regulator of bacteria-phage interactions, but it actually does not play a role in the spatial system with soil. In the spatial model, the external seasonal forcing of the soil temperature gradient plays a dominant role in causing the density of *S* to peak in the warmest season.

For both provinces of Thailand we considered, the spatial model predicts the existence of a pronounced peak of highly virulent phage-free bacteria both near the surface and, even more strikingly, at the depth of approximately $h=30$ cm or deeper. The structure of the bacterial population at shallow depths of soil is affected by seasonal variations of ambient temperature. In the surface water of rice paddies^16^ and in top soil, the *B. pseudomallei* population is predicted to be dominated by the more virulent phage-free bacteria during warmer seasons of the year, whereas during colder winter season phage-associated bacteria are more prevalent. Since surface water and top soil are presumably the main sources of the infection, this is likely to be a major contributing factor to the increase of melioidosis cases during warm seasons in Thailand that is reported in the literature^38^.

The model predicts that the high-density maximum of phage-free bacteria located at around $h=30$ cm would not be affected by the seasonal variation in temperature. The existence of such a peak of phage-free bacterial numbers is somewhat counter-intuitive since one would expect the dominance of lysogenic bacteria at depths characterised by temperatures lower than the critical temperature $T_1$ of transition to the lytic cycles. The observed peak of *S* is a consequence of phage-free bacteria having a higher per-capita effective population growth rate (i.e. replication minus mortality) than of $I_1$. This follows from $\alpha (T) > {\overline{\alpha }}(T)$, as well as from the fact that the transition rate from the lysogenic state to the lytic, with an eventual lysis, is always nonzero. Although at the depth of $h=30$ cm the difference in the effective growth rates is small, this effect accumulates through a large number of generations and *S* eventually outcompetes $I_1$.

Significantly, this prediction is in agreement with environmental sampling data which indicate that *B. pseudomallei* is more frequently found in soil samples taken at 30 cm or deeper, and that bacteria could be found at these depths throughout the year, irrespective of the season^8,9,24^. The existence of a non-seasonal permanent peak of phage-free bacteria at or near $h=30$ cm is important from the pathogen monitoring point of view, as it provides a likely scientific explanation for the empirically derived recommendation that sampling for the presence of *B. pseudomallei* in the environment should be done at the depth of 30 cm^8,9^. For the safety of agricultural workers, the position of the peak of phage-free *B. pseudomallei* in the soil specifies a layer of soil whose disturbance would increase the risk of infection (note that neither lysogenic nor lytic bacteria can cause disease). Coincidentally, 30 cm is the approximate traditional depth of ploughing rice paddy fields. Our study suggests that considering more shallow tillage for rice farming in areas of high endemicity of melioidosis may reduce the risk of infection.

Our model also predicts that enrichment of the environment (e.g. by adding fertiliser) may result in sudden irregular bursts of phage-free *B. pseudomallei* in the upper soil layers, which would be invisible to the human eye. Such blooms occur in narrow soil layers and they can be hard to monitor by standard course sampling, but the density of phage-free bacteria in these layers can be extremely high: a whole order of magnitude higher than at nearby depths. Such layers would present an extra risk of infection for agricultural workers.

From the diagram in Fig. 8, it follows that by increasing mortality of phages $\mu$ (e.g. via the use of agrochemicals that may cause phage mortality, for example those containing ferrous iron) the natural control of bacteria by phages could be affected. A slight increase in phage mortality may make bacteria-phage interactions more regular (seen in the transition from regime B to A). On the other hand, imposing a higher mortality on the phage can eliminate the phage from the soil environment and remove the natural control of *B. pseudomallei*.

In conclusion, our modelling findings reveal that a dominant temperature-responsive clade of phages that is capable of infecting *B. pseudomallei* can control the dynamics of the bacteria and their spatial distribution in the soil environment. Taking into account our modelling outcomes can potentially help to improve current melioidosis prevention efforts in Southeast Asia and across the world.

## Supplementary Information

Supplementary Information.
