# Supplementary material for: Modelling the spatiotemporal complexity of interactions between pathogenic bacteria and a phage with a temperature-dependent life cycle switch
Source: Sci Rep. 2021 Feb 23;11:4382. doi: 10.1038/s41598-021-83773-1 (PMC7902855; doi:10.1038/s41598-021-83773-1)
Supplement: Supplementary file 1 — Supplementary Information. [file 41598_2021_83773_MOESM1_ESM.pdf]

Supplementary Materials for the manuscript

‘Modelling the spatiotemporal complexity of interactions between pathogenic bacteria and a phage with a temperature-dependent life cycle switch’

by Halil I. Egilmez[1], Andrew Yu. Morozov[2,3*] and Edouard E. Galyov[2]

[1]Hitit University, Osmancik MYO, 19500, Corum, Turkey

[2]University of Leicester, University Rd, Leicester, LE1 7RH, United Kingdom

[3]Institute of Ecology and Evolution, Russian Academy of Sciences, 119071, Moscow, Russia

*corresponding author: am379@leicester.ac.uk

In the supplementary material we provide extra model simulations of bacteria-phage dynamics which are not included in the main text. We also provide detail on fitting the dependence of the carrying capacity of bacteria on depth.

Table of Contents

Estimating the dependence of the carrying capacity on the depth (SM1)… 2

Seasonal variation of temperature in the soil predicted by the model (SM2)…....................... 3

Vertical profiles of infected bacteria and phages in Nakhon Phanom province (SM3) 5

Influence of C_{surf} on vertical profiles of bacteria and phages in Nakhon Phanom (SM4)........ 9

Influence of D_b, D_p on vertical profiles of bacteria and phages in Nakhon Phanom (SM5)…12

Spatiotemporal dynamics predicted for Sa Kaeo province (SM6)… 28

SM1

We parametrise the dependence of the carrying capacity of the *B. pseudomallei* population on depth in the soil. We firstly reveal the dependence of the overall bacterial load on depth in paddy soils in Southern China using the data from Wang et al 2017. Note that the considered data set includes a large number of bacterial strains. We use the Gaussian parameterisation curve in the form of $C(h) = (C_{surf}^0 - C_0^0)\exp(-Bh^2) + C_0^0$. We fix the value of C_0^0 to be $3 \times 10^8 cell/\mathrm{ml}^{-1}$ which matches the bacterial numbers at large depths $h \geq 100cm$ (fixing C_0^0 is needed to reduce the number of parameters for a small overall number of data points). The other two coefficients can be easily estimated via the non-linear regression procedure using GraphPad Prism software. Our parameter fit provides the following estimates: $C_{\mathrm{surf}}^0 = (2.2 \pm 0.5) \times 10^{10}\ cell/\mathrm{ml}^{-1}$, $B = 7.5\ \pm 0.4 \times 10^{-4}\ 1/cm^2$. Note that directly fitting 3 parameters to the data gives close results for the estimates, however, with larger errors. The corresponding fit and the date are shown in Figure 1S(a).

The next step is to re-scale the obtained curve using the field study of Trung et al 2011, where the abundance of *B. pseudomallei* in highly endemic areas of Thailand was reported to reach values from 1×10^5 to 8×10^5 cells per gram of soil at the depth of 30 cm. Thus we can conclude that using the rescaled values $C_{surf} = 10^6\ cell/\mathrm{ml}^{-1}$ and $C_0 = 10^4\ cell/\mathrm{ml}^{-1}$ will provide a good agreement with empirical data (we assume that the proposed re-scaling is maintained at different depths of soil and not only at *h*=30cm). The corresponding graph of *C*(*h*) for the default values of parameters is shown in Figure 1S(b). Note that the estimate of C_{surf} is also with agreement with our studies (e.g. Göhler et al., 2017).

(a)

(b)

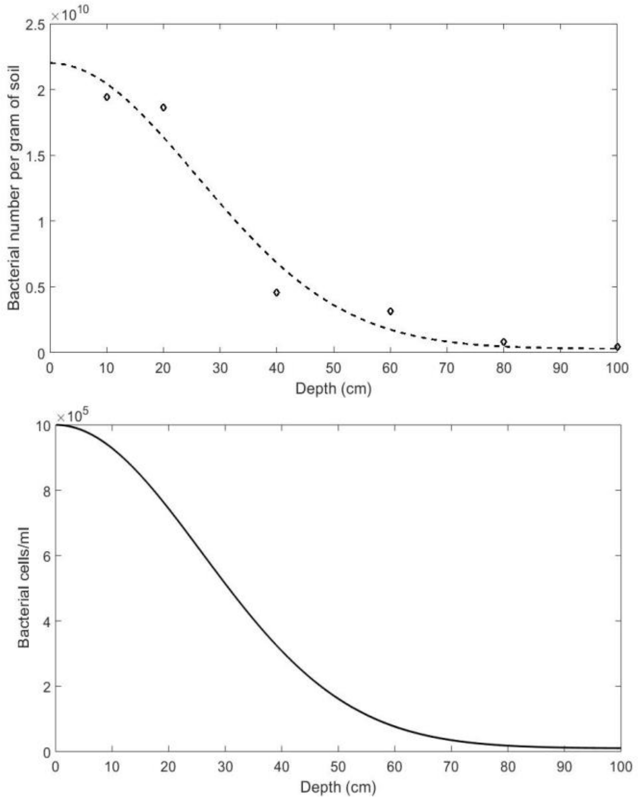

Figure 1S (a): Fitting the curve to the empirical data of the vertical dependence of the total bacterial load in paddy soil from the study by Wang et al 2017 (Plos One, 12, e0189506). The bacterial numbers correspond to the data collected in Yingtan region (China). (**b**)**:** Carrying capacity *C*(*h*) for total *B. pseudomallei* plotted for the default values of model parameters $C_{surf} = 10^6\ cell/ml$; $B = 7.5\ \times 10^{-4}\ 1/cm^2$; $C_0 = 10^4\ cell/ml$.

SM2

In Figures 2S-3S we show vertical distributions of temperature in the soil across the four seasons predicted by the model. The considered province of Thailand is Nakhon Phanom. The model parameters are taken as default values from Table 1.

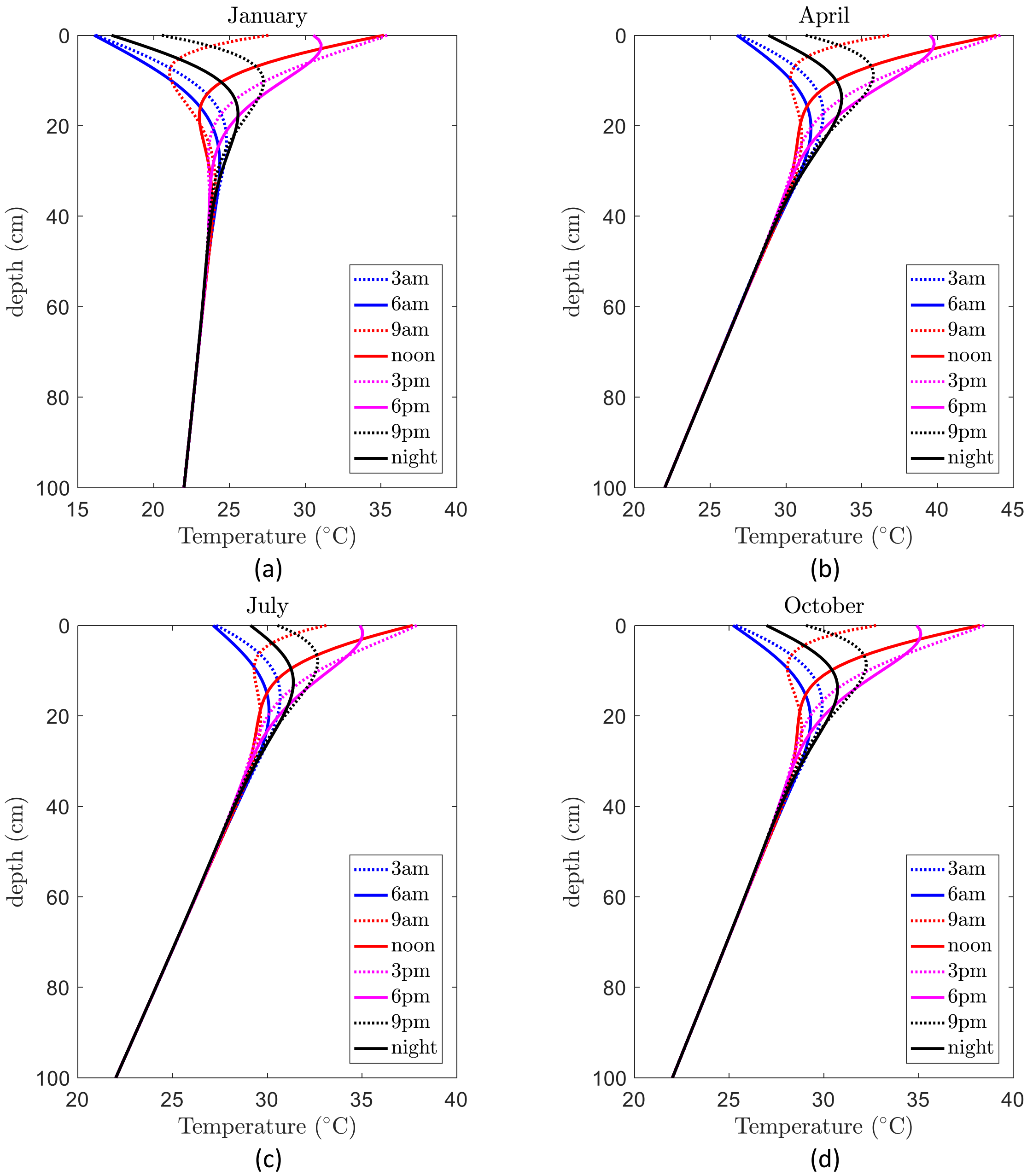

Figure 2S: Vertical distributions of the temperature *T* across the soil in a typical field in Nakhon Phanom province in Thailand predicted by heat equation (2) using historical data for the surface temperatures for the period of 2013-2016. Each panel represents the first day of the following months: January (a); April (b); July (c) and October (d); the vertical profiles are shown for every 3 hours.

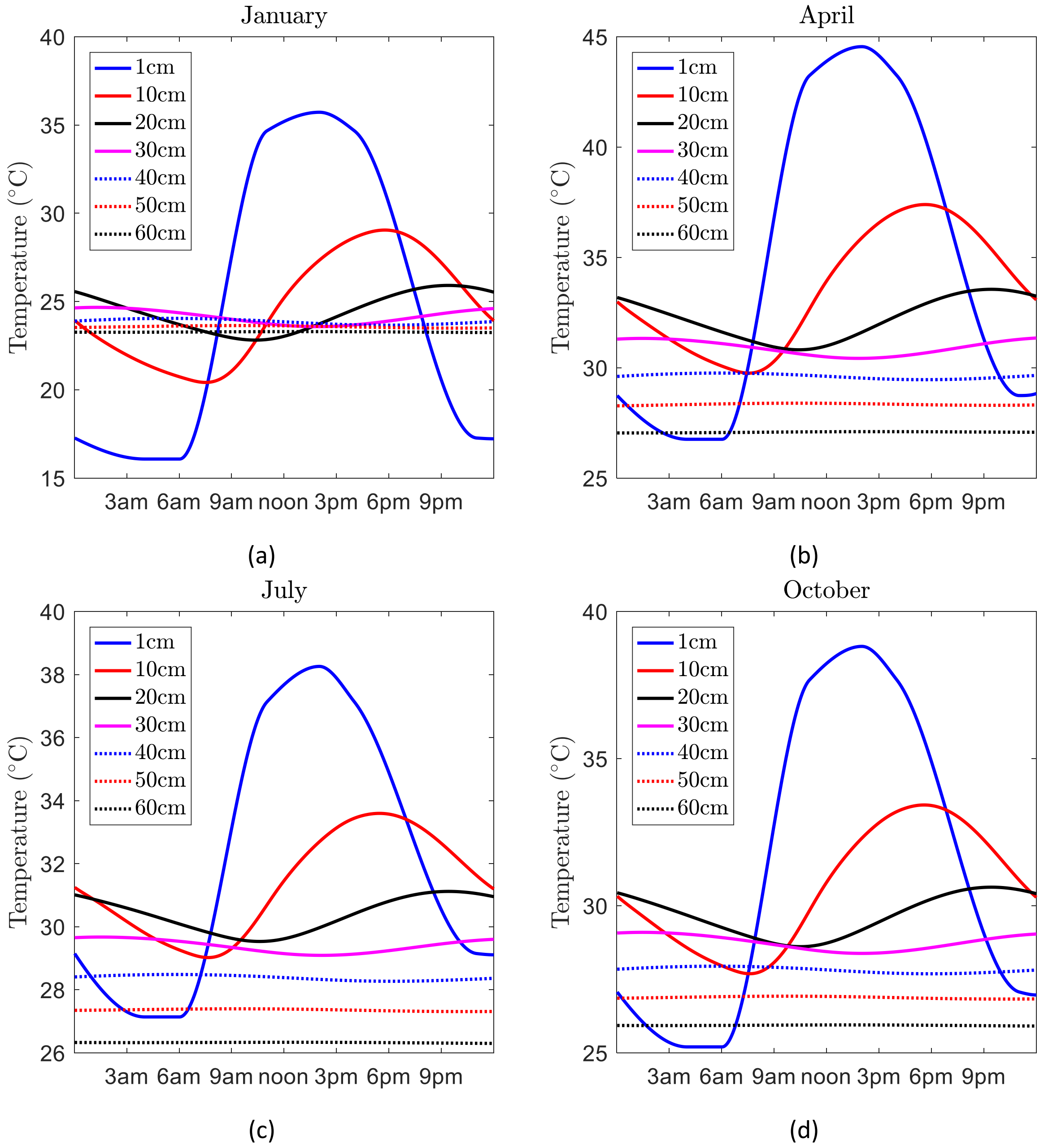

Figure 3S: Daily temperature variation at a fixed depth of the soil in Nakhon Phanom province predicted by equation (2) using historical data for the surface temperatures for the period of 2013-2016. Each panel represents the first day of the following months: January (a); April (b); July (c) and October (d).

SM3

In Figures 4S-7S we show vertical distributions of infected bacteria and phages in the soil across the four seasons predicted by the model. The considered province of Thailand is Nakhon Phanom. The model parameters are taken as default values from Table 1.

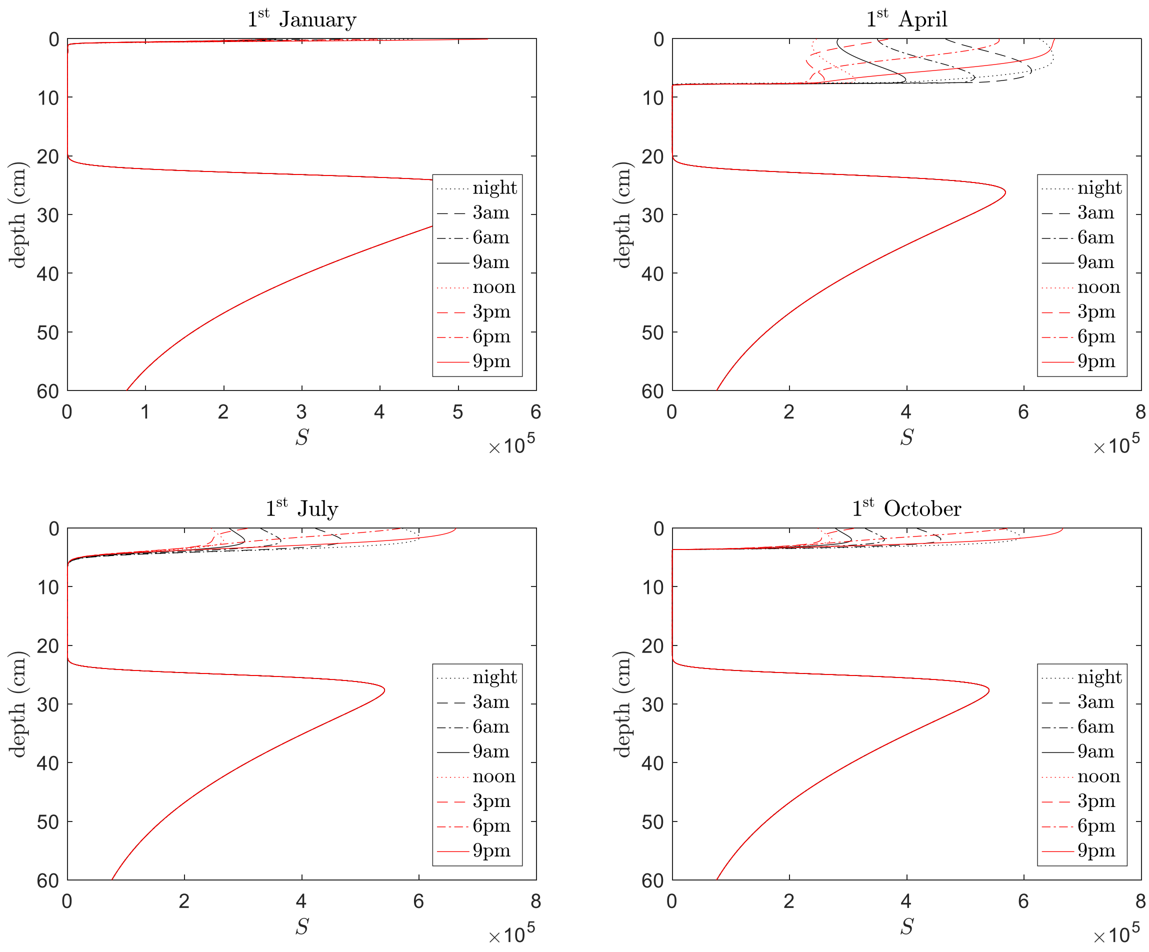

Figure 4S: Vertical distributions of susceptible bacteria *S* in the soil throughout the day for four seasons predicted by the model calculated for Nakhon Phanom province. Time of the day is indicated in the box of each figure. Model parameters are taken from Table 1 as default values. The unit of the density of *S* is cell/ml.

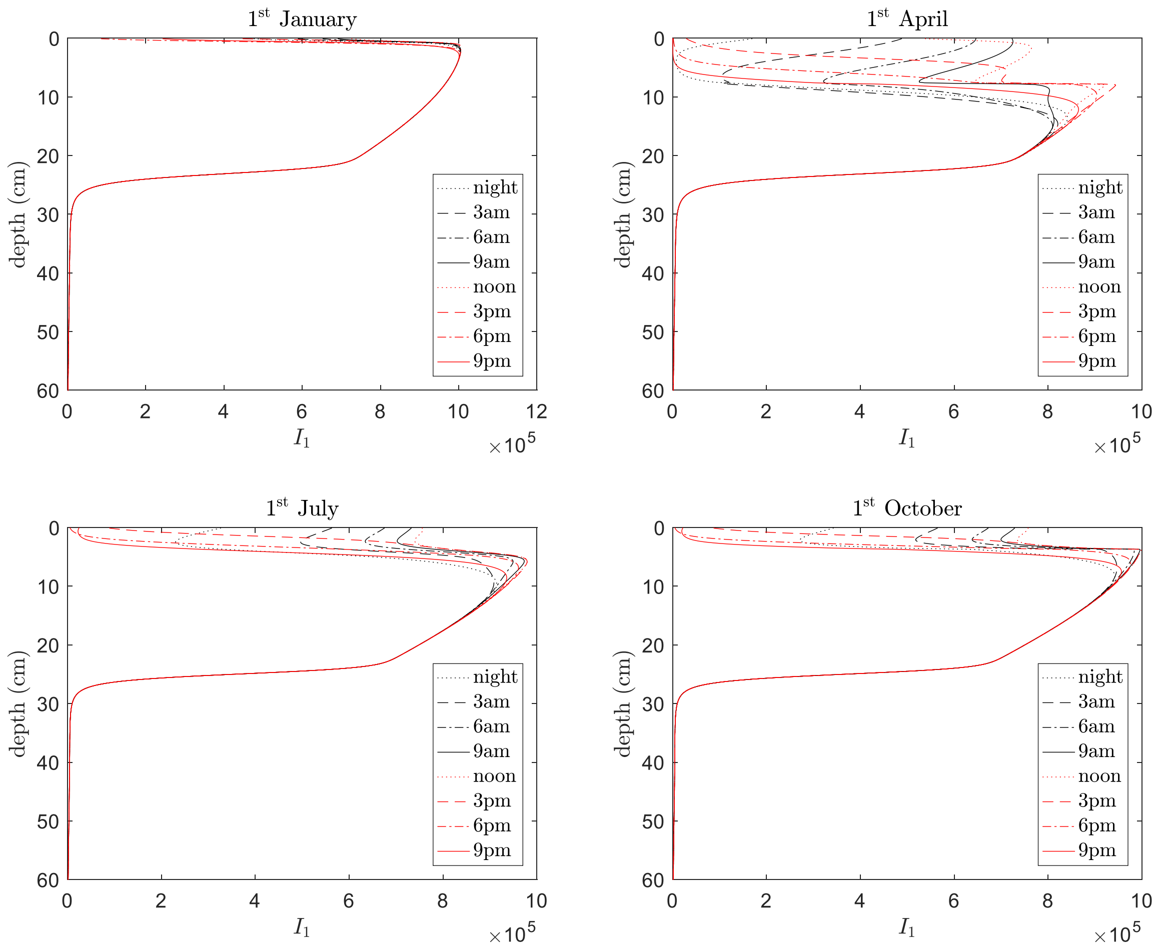

Figure 5S: Vertical distributions of infected bacteria in lysogenic stage I_1 in the soil throughout the day for four seasons predicted by the model calculated for Nakhon Phanom province. Time of the day is indicated in the box of each figure. Model parameters are taken from Table 1 as default values. The unit of the density of I_1 is cell/ml.

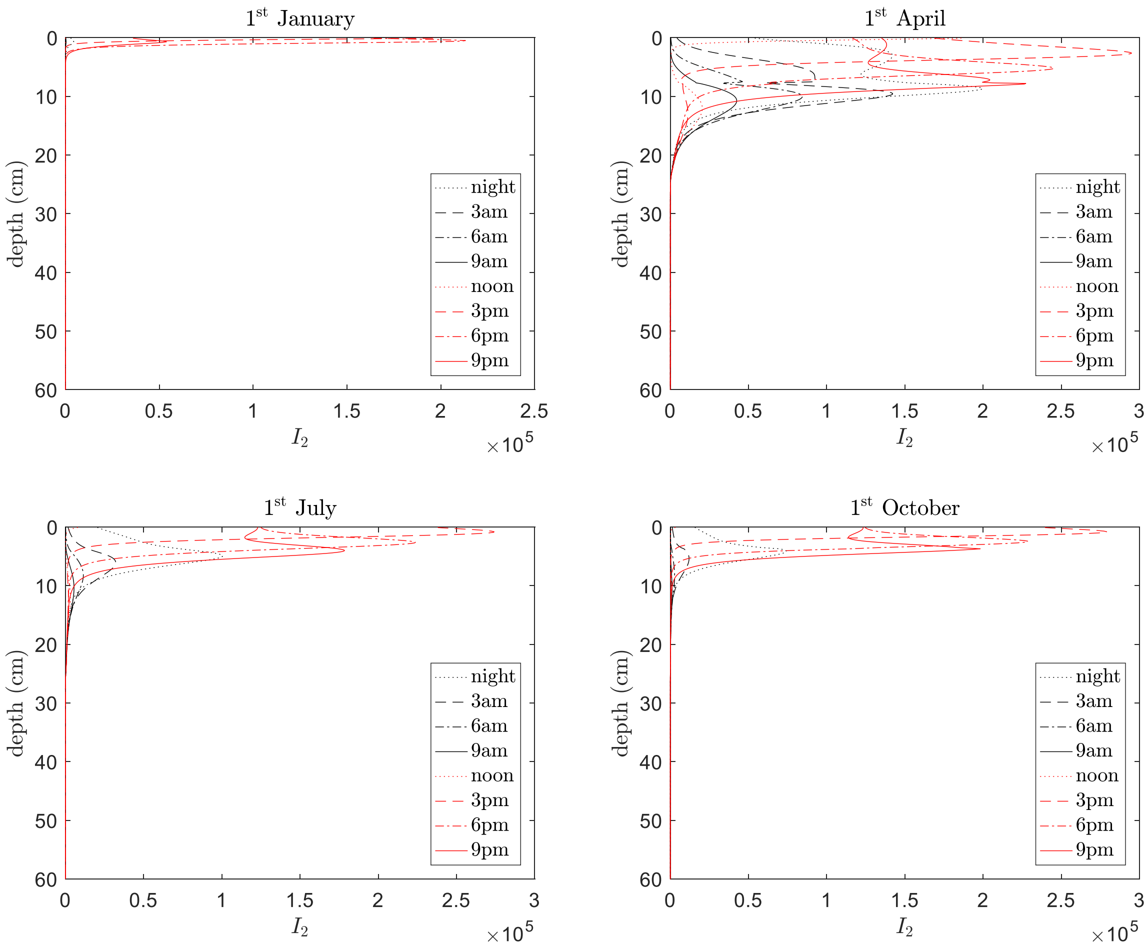

Figure 6S: Vertical distributions of infected bacteria in lytic stage I_2 in the soil throughout the day for four seasons predicted by the model calculated for Nakhon Phanom province. Time of the day is indicated in the box of each figure. Model parameters are taken from Table 1 as default values. The unit of the density of I_2 is cell/ml.

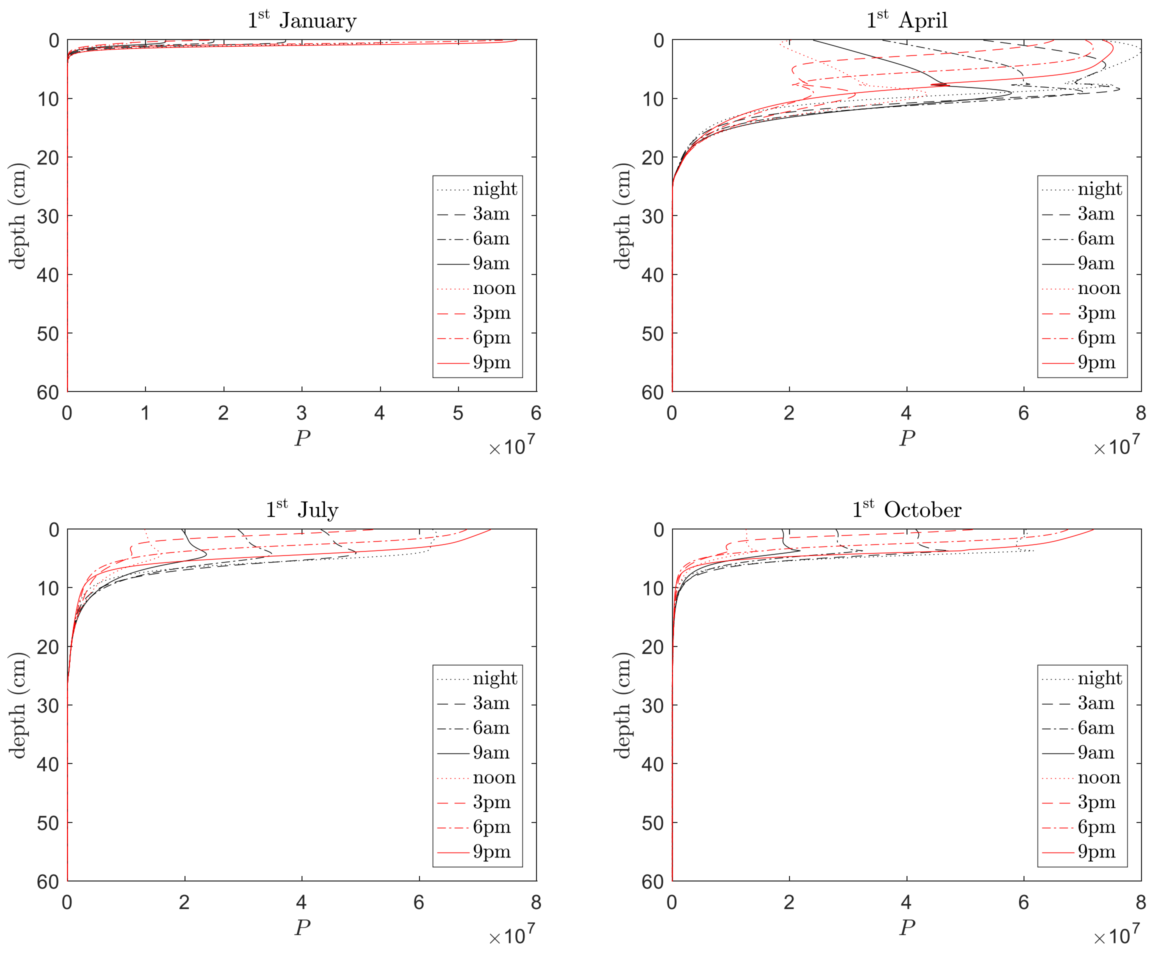

Figure 7S: Vertical distributions of the phage *P* in the soil throughout the day for four seasons predicted by the model calculated for Nakhon Phanom province. Time of the day is indicated in the box of each figure. Model parameters are taken from Table 1 as default values. The unit of the density of is phage/ml.

SM4

In Figures 8S-10S we show the influence of vertical distributions of infected bacteria and phages on the carrying capacity C_{surf}. The other model parameters are as the default values from Table 1. The spatial distributions are obtained for on April 1st. The considered province of Thailand is Nakhon Phanom.

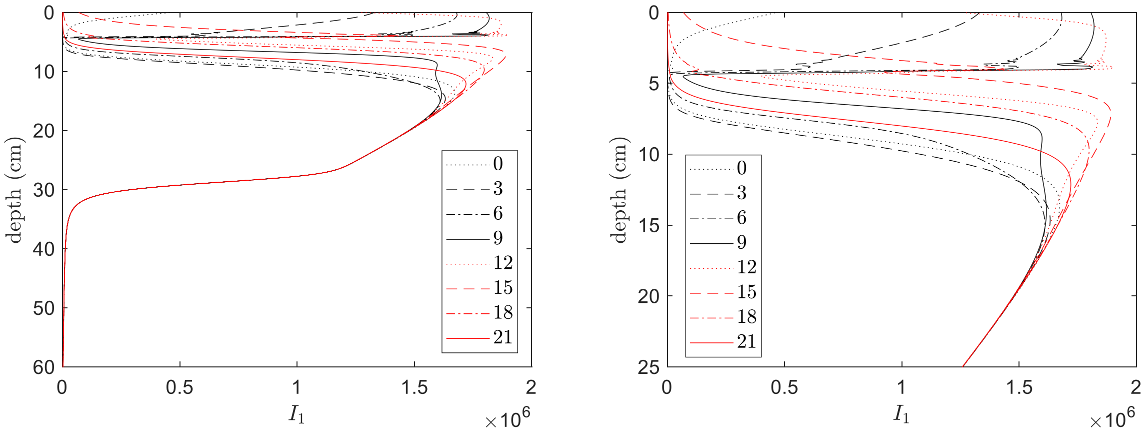

(a) $C_{\text{surf}} = 2 * 10^6$ cell/ml

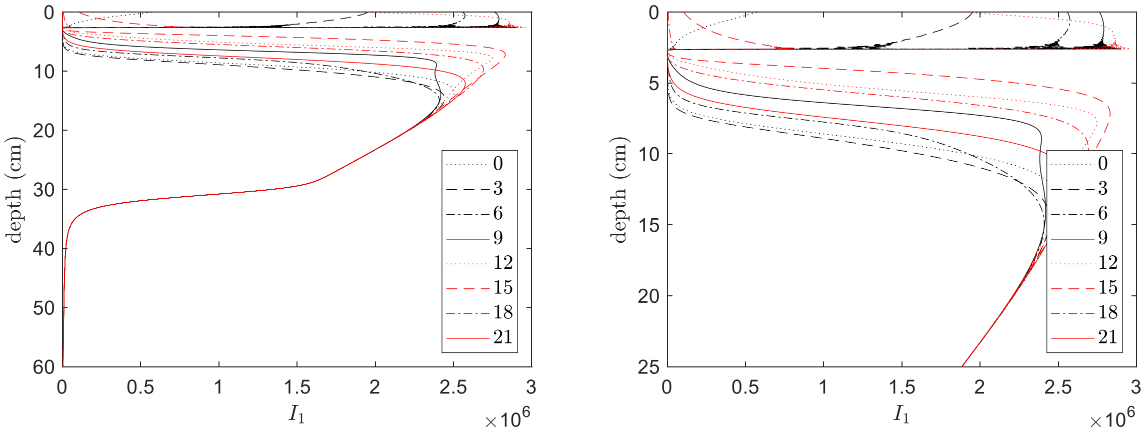

(b) $C_{\text{surf}} = 3 * 10^6$ cell/ml

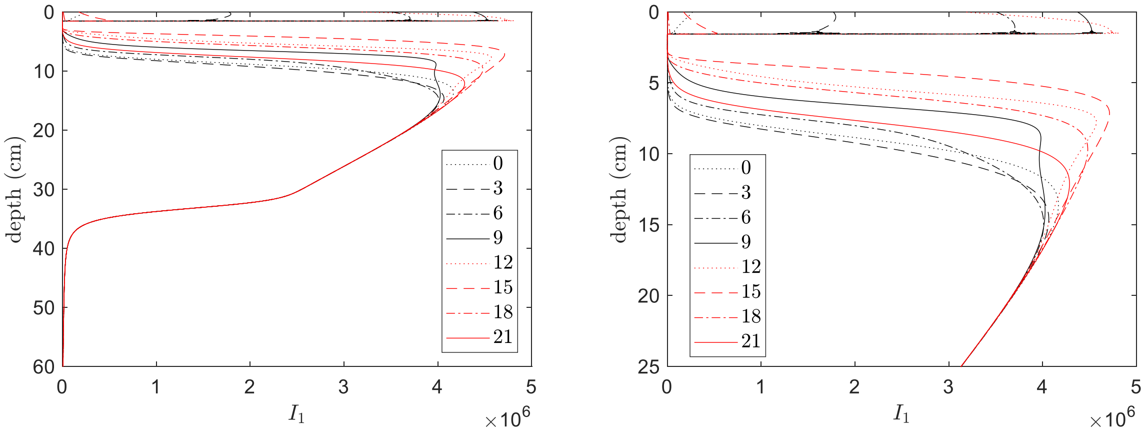

(c) $C_{\text{surf}} = 5 * 10^6$ cell/ml

Figure 8S. Vertical distributions of infected bacteria in lysogenic stage I_1 in the soil for different values of C_{surf}. Time of the day is indicated in the box of each figure. The left panel shows vertical distributions of I_1 in the top 60 cm whereas the right panel presents zooms of the same profiles near the surface. The unit of the density of I_1 is cell/ml.

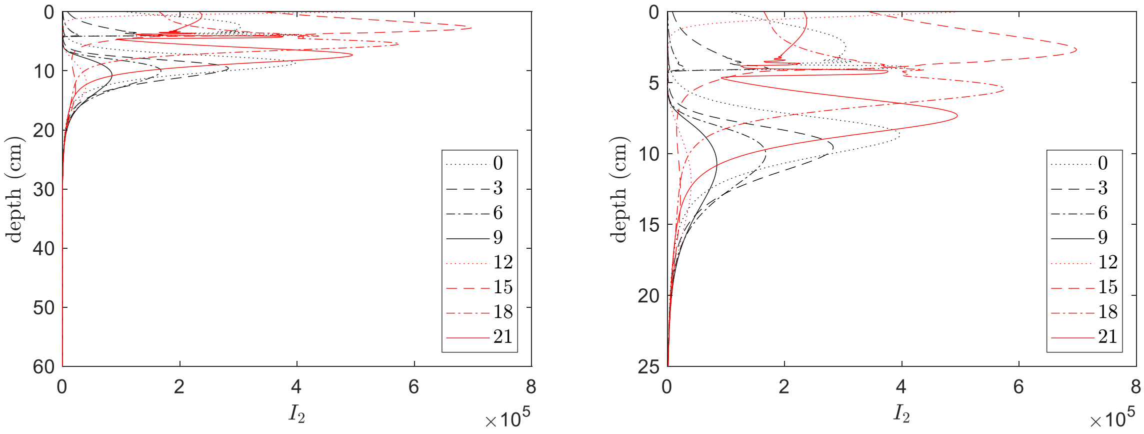

(a) $C_{\mathrm{surf}} = 2 * 10^6$ cell/ml

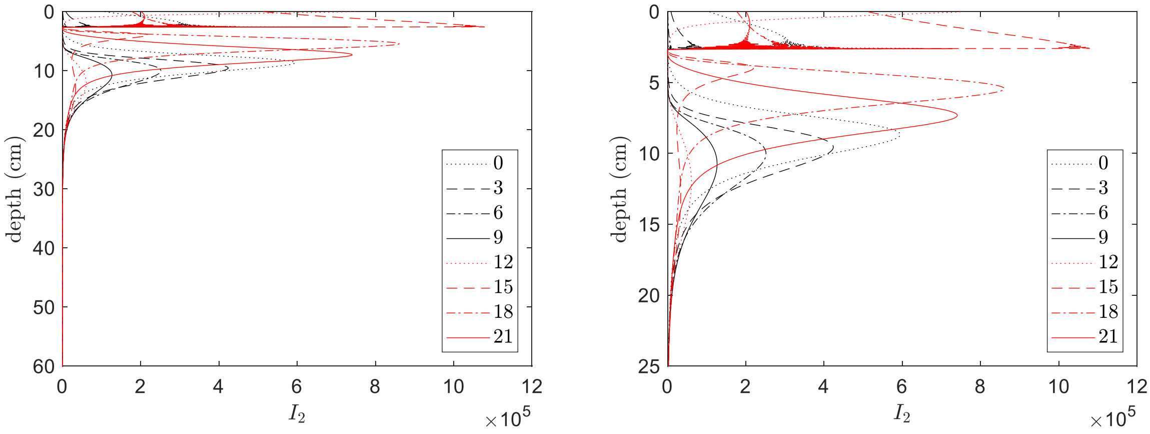

(b) $C_{\mathrm{surf}} = 3 * 10^6$ cell/ml

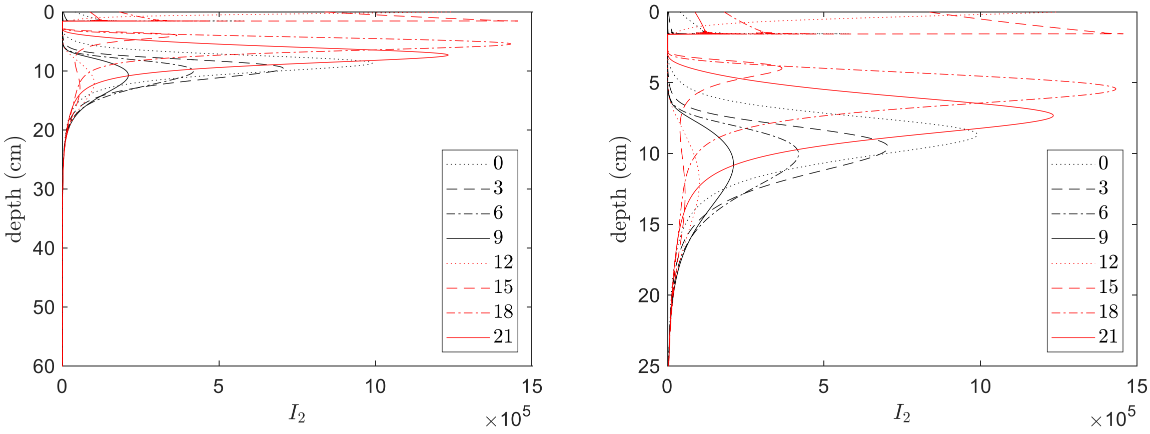

(c) $C_{\mathrm{surf}} = 5 * 10^6$ cell/ml

Figure 9S. Vertical distributions of infected bacteria in lysogenic stage I_2 in the soil for different values of C_{surf}. Time of the day is indicated in the box of each figure. The left panel shows vertical distributions of I_2 in the top 60 cm whereas the right panel presents zooms of the same profiles near the surface. The unit of the density of I_2 is cell/ml.

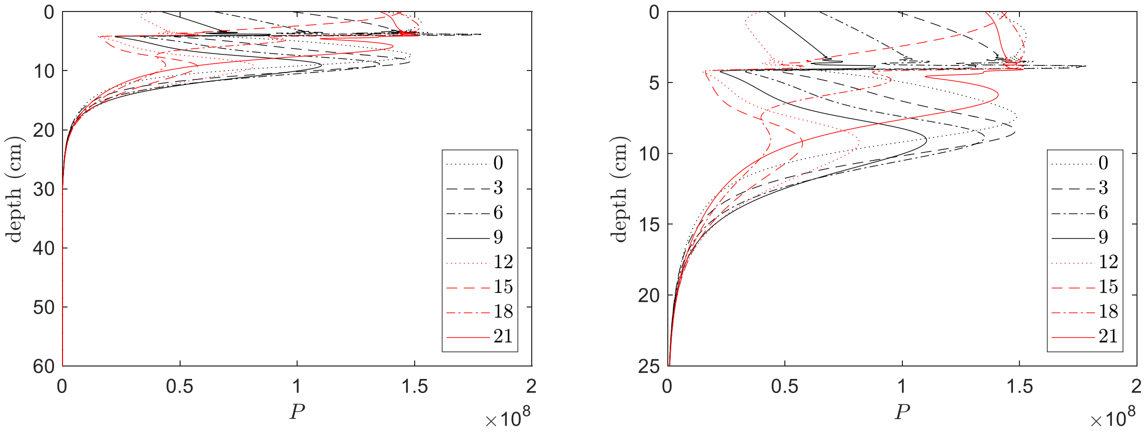

(a) $C_{\text{surf}} = 2 * 10^6$ cell/ml

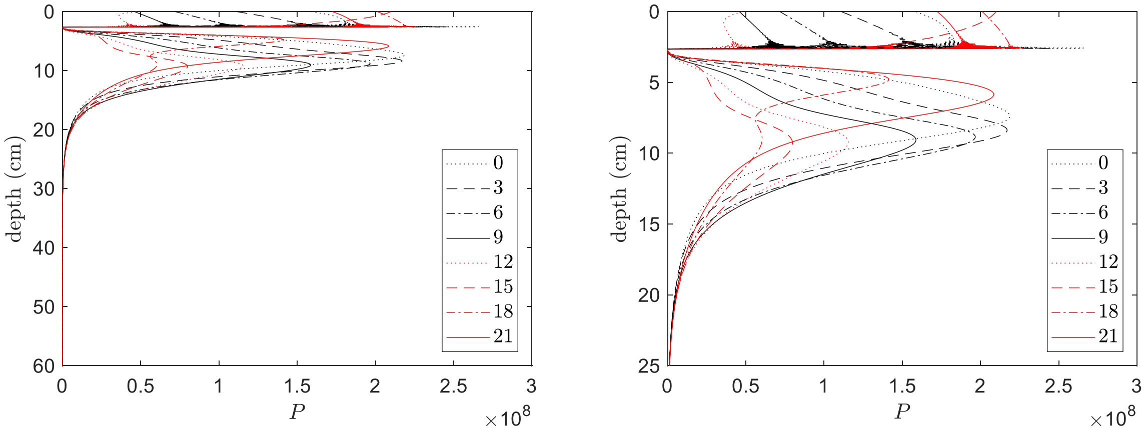

(b) $C_{\text{surf}} = 3 * 10^6$ cell/ml

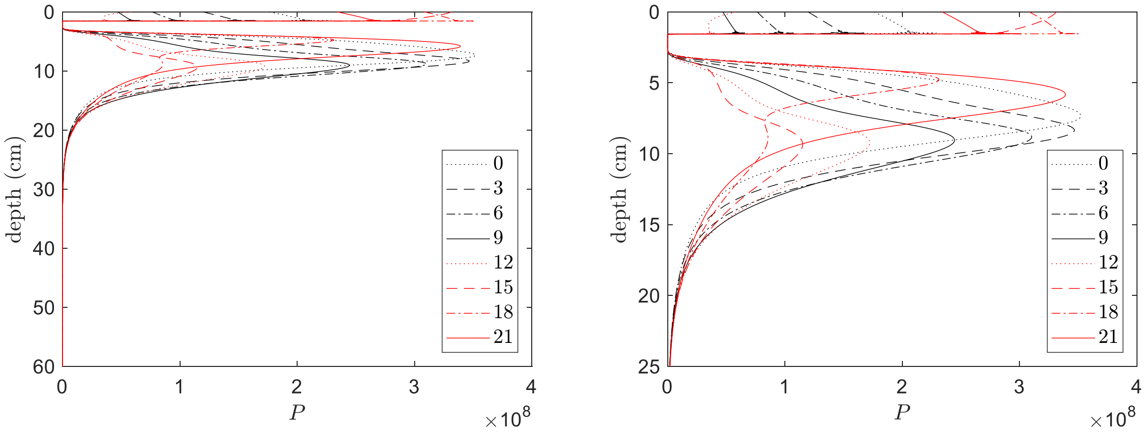

(c) $C_{\text{surf}} = 5 * 10^6$ cell/ml

Figure 10S. Vertical distributions of free phages P in the soil for different values of C_{surf}. Time of the day is indicated in the box of each figure. The left panel shows vertical distributions of P in the top 60 cm whereas the right panel presents zooms of the same profiles near the surface. The unit of the density of P is phages/ml.

SM5

In Figures 11S-26S we show the effects of variation of the diffusion coefficients D_p and D_b on the vertical distributions of bacteria and phages. The spatial distributions are obtained for the four different seasons of the year.

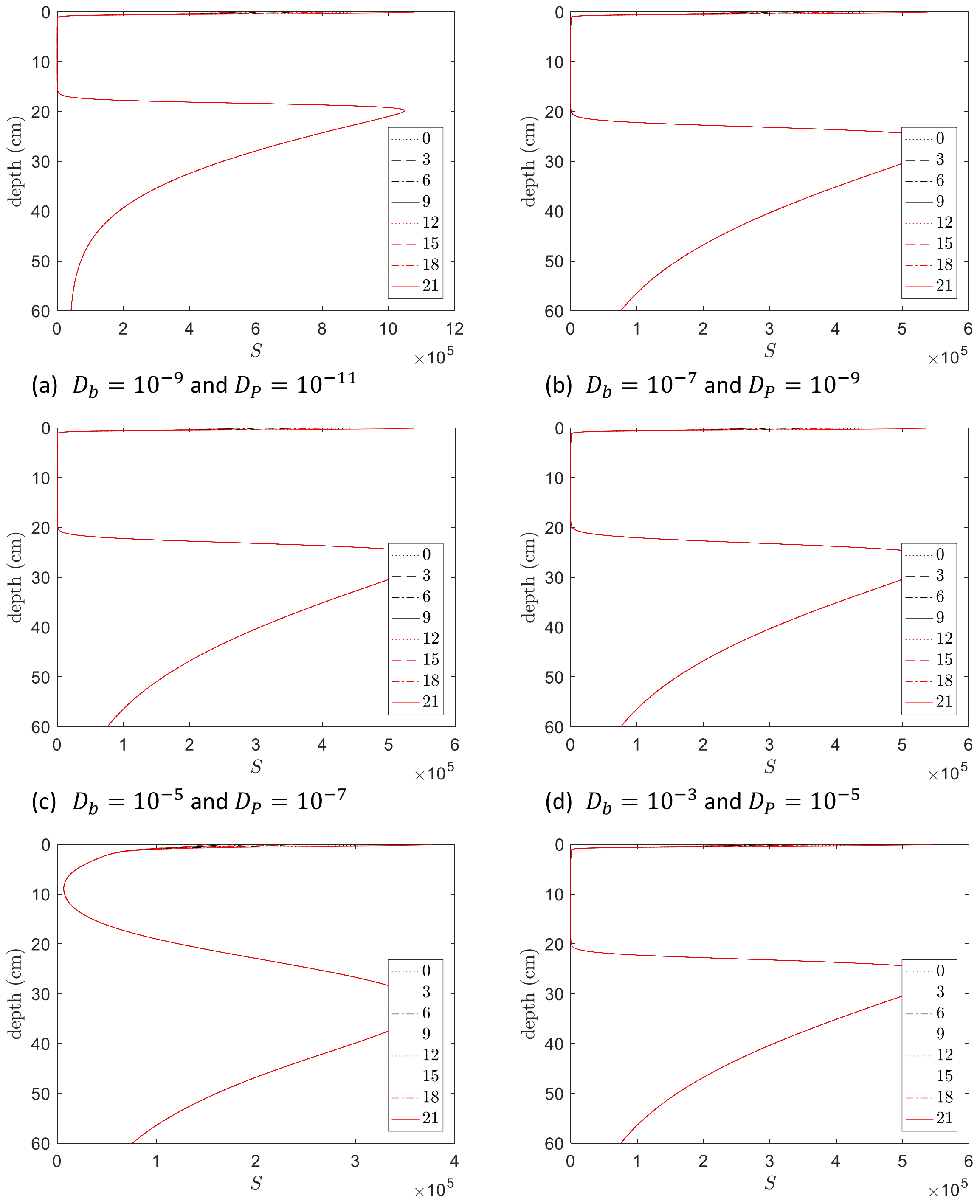

(a) $D_b = 10^{-9}$ and $D_P = 10^{-11}$

(b) $D_b = 10^{-7}$ and $D_P = 10^{-9}$

(c) $D_b = 10^{-5}$ and $D_P = 10^{-7}$

(d) $D_b = 10^{-3}$ and $D_P = 10^{-5}$

(e) $D_b = 10^{-1}$ and $D_P = 10^{-3}$

(f) $D_b = 10^{-7}$ and $D_P = 10^{-2}$

Figure 11S. Vertical distributions of susceptible bacteria S in the soil throughout the day on January 1st (Nakhon Phanom province) for different D_p and D_b measured in cm^2/day. Time of the day is indicated in the box of each figure. Model parameters are taken from Table 1 as default values. The unit of the density of S is cell/ml.

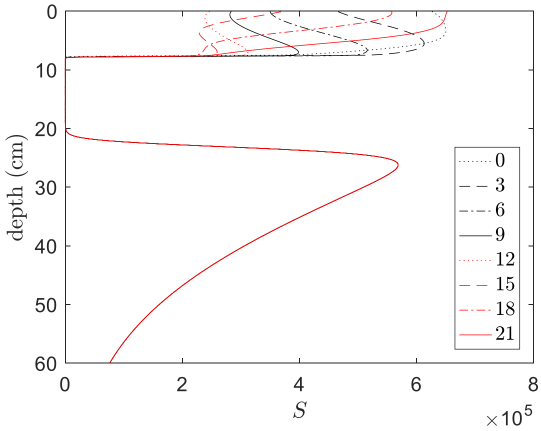

(a) $D_b = 10^{-9}$ and $D_P = 10^{-11}$

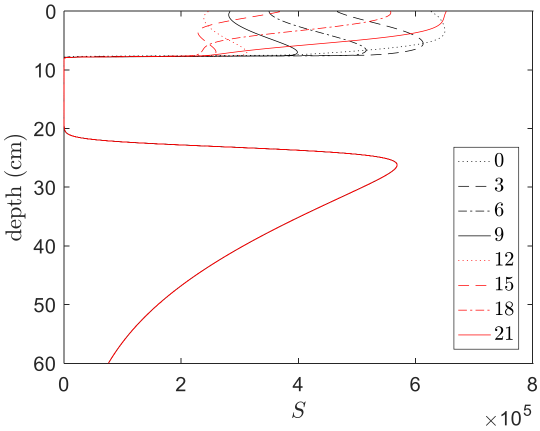

(b) $D_b = 10^{-7}$ and $D_P = 10^{-9}$

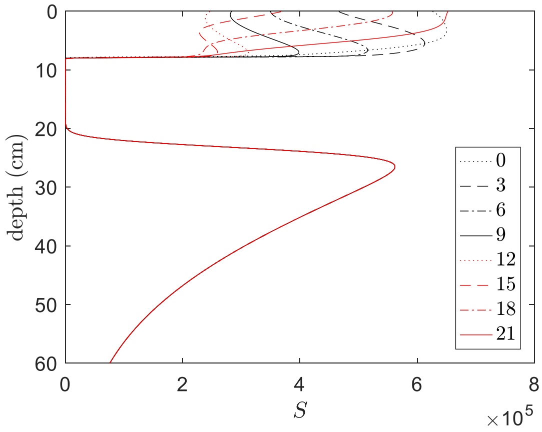

(c) $D_b = 10^{-5}$ and $D_P = 10^{-7}$

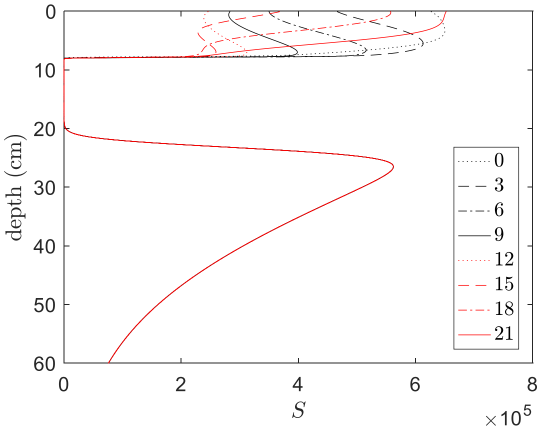

(d) $D_b = 10^{-3}$ and $D_P = 10^{-5}$

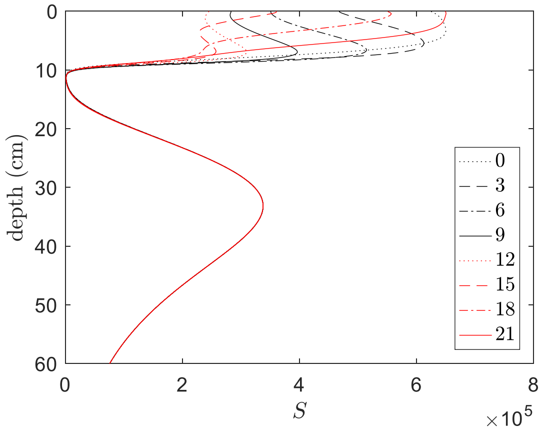

(e) $D_b = 10^{-1}$ and $D_P = 10^{-3}$

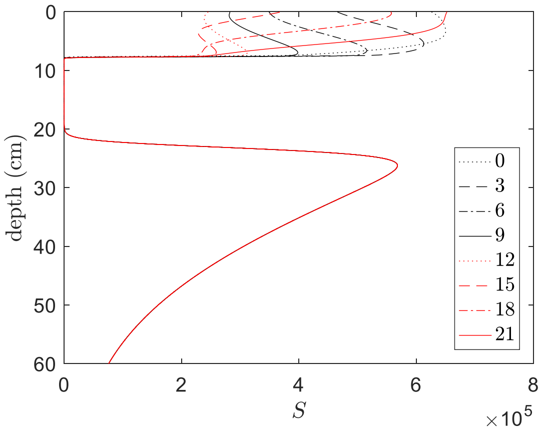

(f) $D_b = 10^{-7}$ and $D_P = 10^{-2}$

Figure 12S. Vertical distribution of susceptible bacteria S in the soil a throughout the day on April 1st (Nakhon Phanom province) for different D_p and D_b measured in cm^2/day. Time of the day is indicated in the box of each figure. Model parameters are taken from Table 1 as default values. The unit of the density of S is cell/ml.

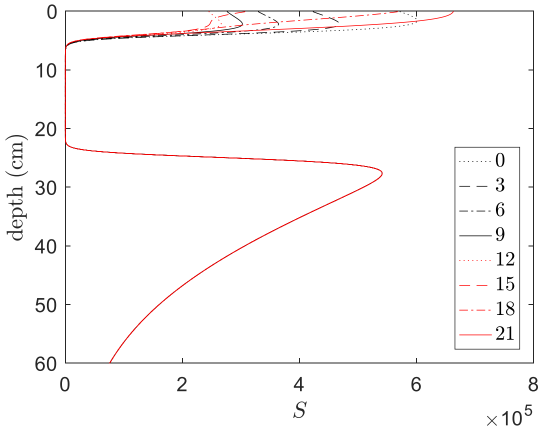

(a) $D_b = 10^{-9}$ and $D_P = 10^{-11}$

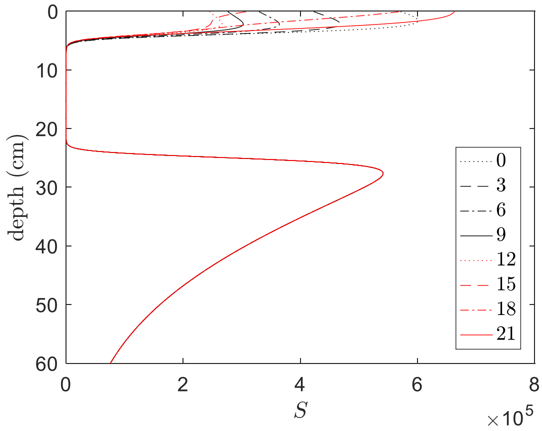

(b) $D_b = 10^{-7}$ and $D_P = 10^{-9}$

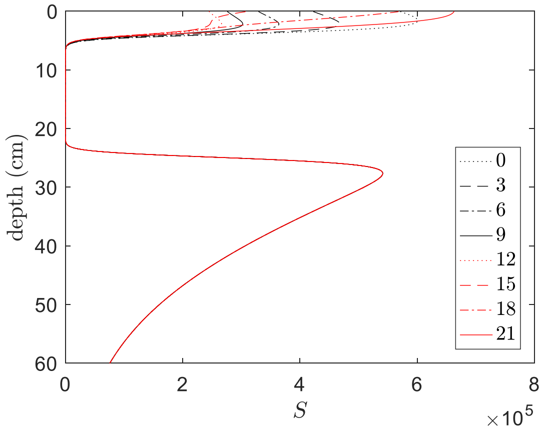

(c) $D_b = 10^{-5}$ and $D_P = 10^{-7}$

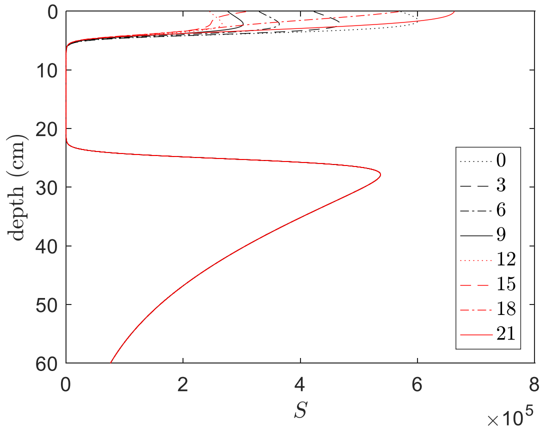

(d) $D_b = 10^{-3}$ and $D_P = 10^{-5}$

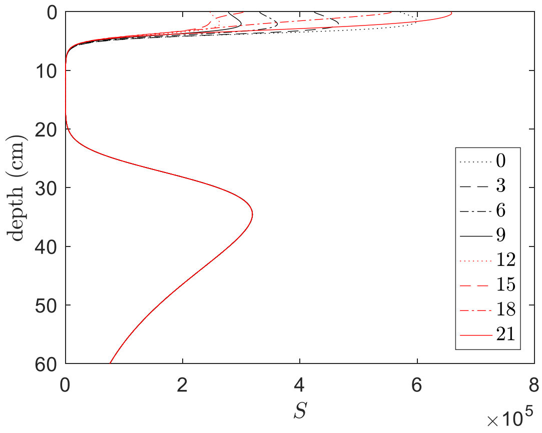

(e) $D_b = 10^{-1}$ and $D_P = 10^{-3}$

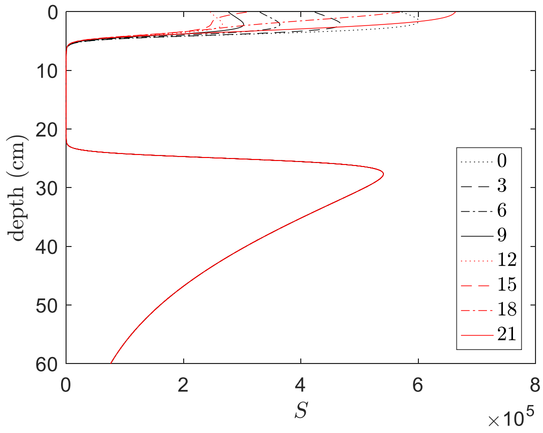

(f) $D_b = 10^{-7}$ and $D_P = 10^{-2}$

Figure 13S. Vertical distribution of susceptible bacteria S in the soil throughout the day on July 1st (Nakhon Phanom province) for different D_p and D_b measured in cm^2/day. Time of the day is indicated in the box of each figure. Model parameters are taken from Table 1 as default values. The unit of the density of S is cell/ml.

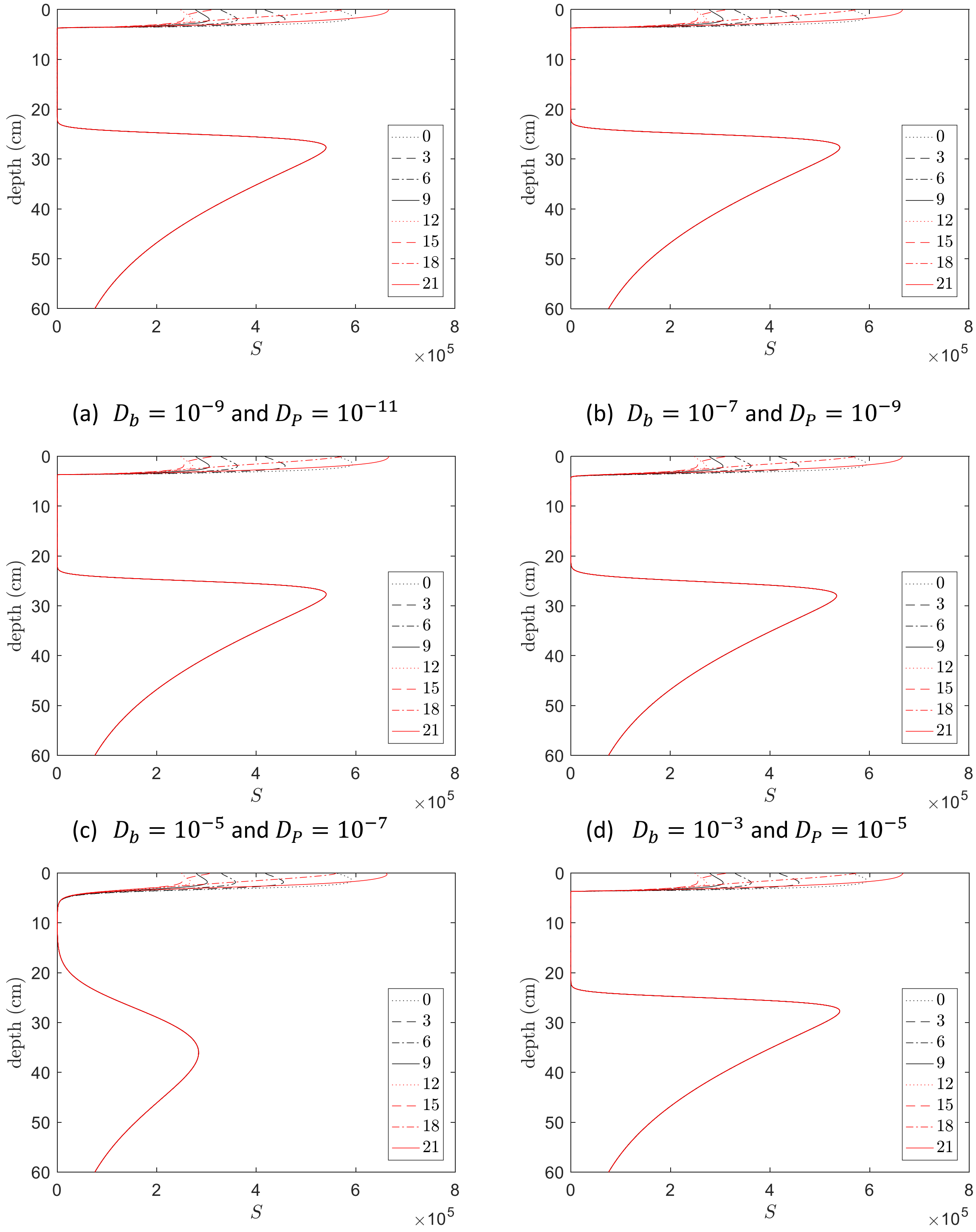

(a) $D_b = 10^{-9}$ and $D_P = 10^{-11}$

(b) $D_b = 10^{-7}$ and $D_P = 10^{-9}$

(c) $D_b = 10^{-5}$ and $D_P = 10^{-7}$

(d) $D_b = 10^{-3}$ and $D_P = 10^{-5}$

(e) $D_b = 10^{-1}$ and $D_P = 10^{-3}$

(f) $D_b = 10^{-7}$ and $D_P = 10^{-2}$

Figure 14S. Vertical distribution of susceptible bacteria S in the soil throughout the day on October 1st (Nakhon Phanom province) for different D_p and D_b measured in cm^2/day. Time of the day is indicated in the box of each figure. Model parameters are taken from Table 1 as default values. The unit of the density of S is cell/ml.

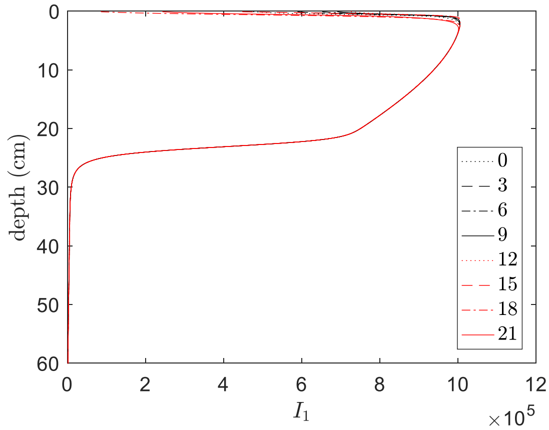

(a) $D_b = 10^{-9}$ and $D_P = 10^{-11}$

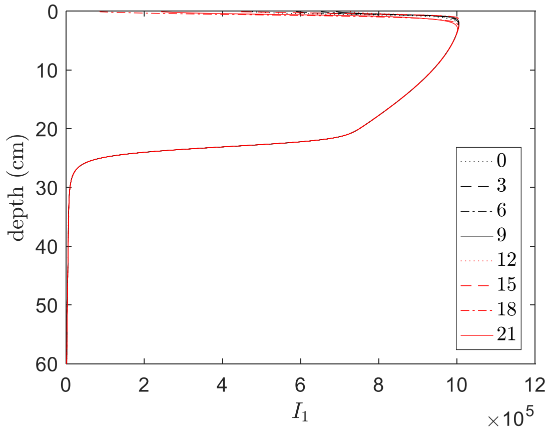

(b) $D_b = 10^{-7}$ and $D_P = 10^{-9}$

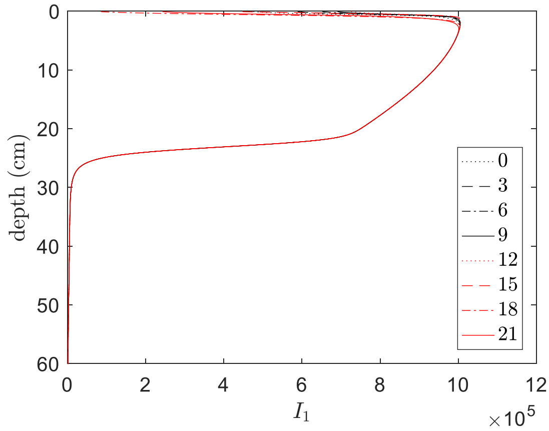

(c) $D_b = 10^{-5}$ and $D_P = 10^{-7}$

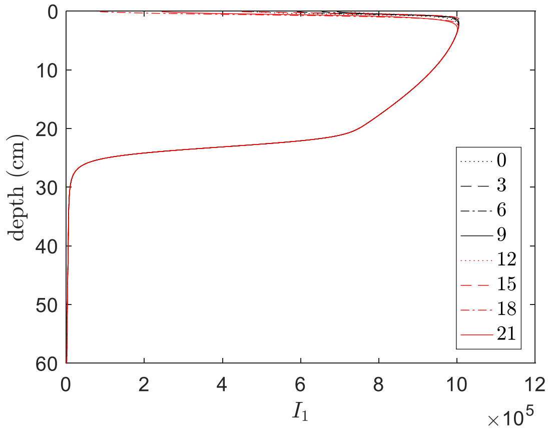

(d) $D_b = 10^{-3}$ and $D_P = 10^{-5}$

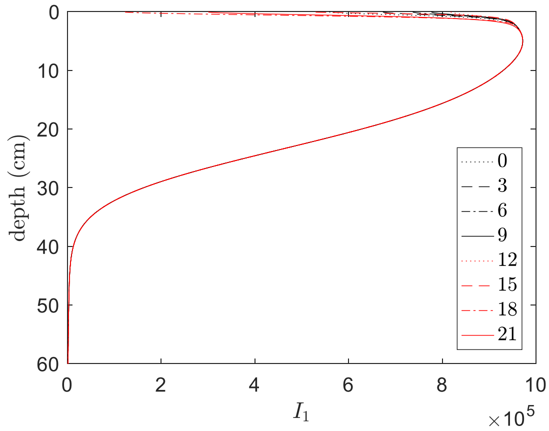

(e) $D_b = 10^{-1}$ and $D_P = 10^{-3}$

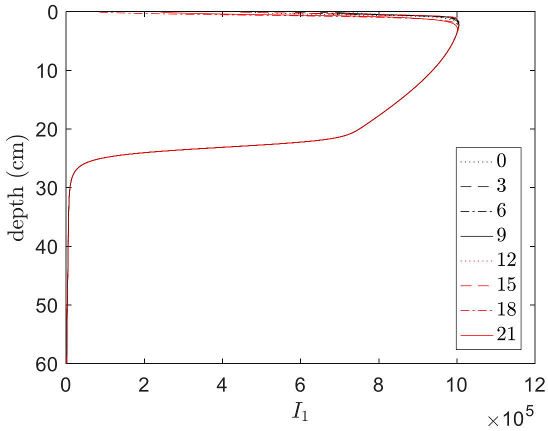

(f) $D_b = 10^{-7}$ and $D_P = 10^{-2}$

Figure 15S. Vertical distributions of infected bacteria in lysogenic stage I_1 in the soil throughout the day on January 1st (Nakhon Phanom province) for different D_p and D_b measured in cm^2/day. Time of the day is indicated in the box of each figure. Model parameters are taken from Table 1 as default values. The unit of the density of I_1 is cell/ml.

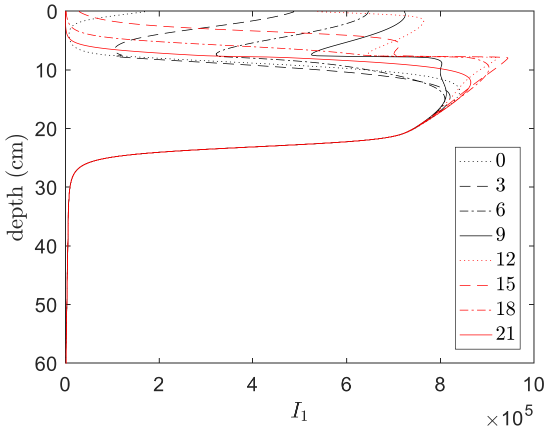

(a) $D_b = 10^{-9}$ and $D_P = 10^{-11}$

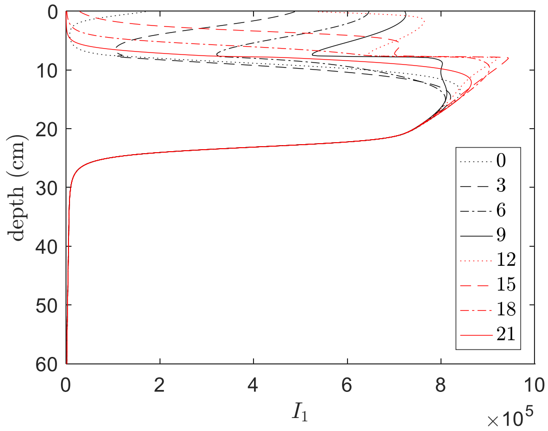

(b) $D_b = 10^{-7}$ and $D_P = 10^{-9}$

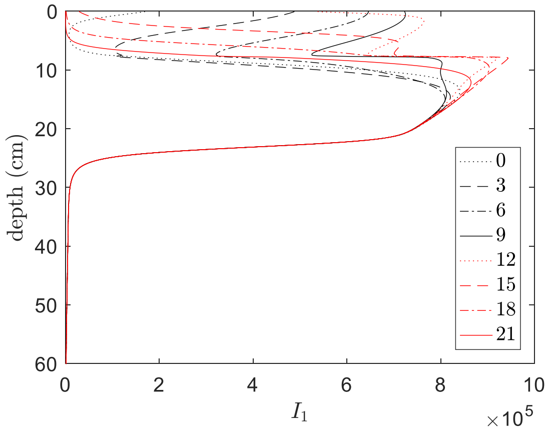

(c) $D_b = 10^{-5}$ and $D_P = 10^{-7}$

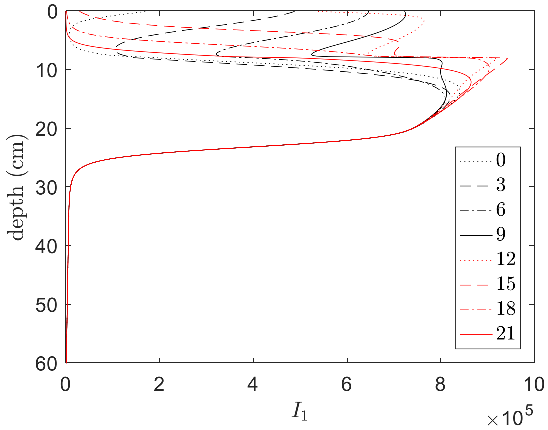

(d) $D_b = 10^{-3}$ and $D_P = 10^{-5}$

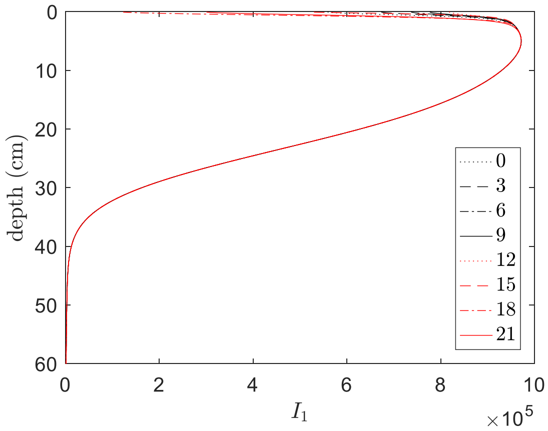

(e) $D_b = 10^{-1}$ and $D_P = 10^{-3}$

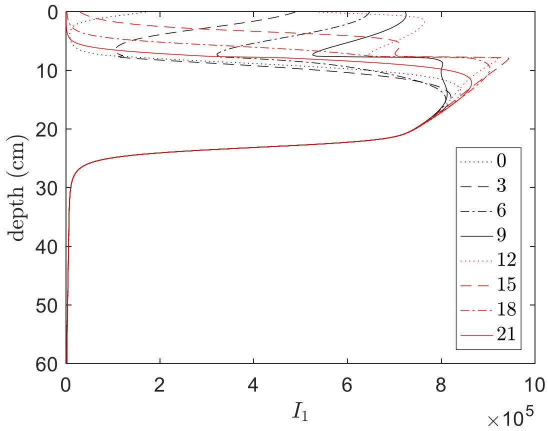

(f) $D_b = 10^{-7}$ and $D_P = 10^{-2}$

Figure 16S. Vertical distributions of infected bacteria in lysogenic stage I_1 in the soil throughout the day on April 1st (Nakhon Phanom province) for different D_p and D_b measured in cm^2/day. Time of the day is indicated in the box of each figure. Model parameters are taken from Table 1 as default values. The unit of the density of I_1 is cell/ml.

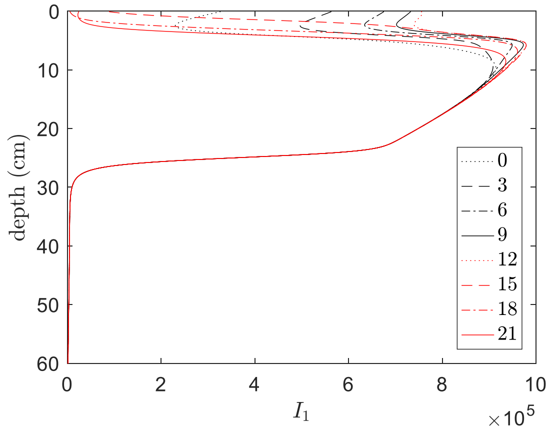

(a) $D_b = 10^{-9}$ and $D_P = 10^{-11}$

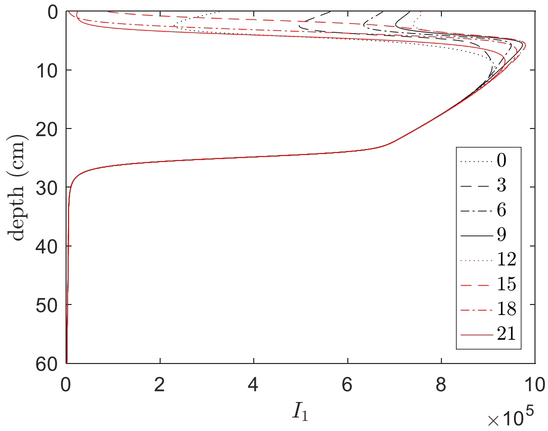

(b) $D_b = 10^{-7}$ and $D_P = 10^{-9}$

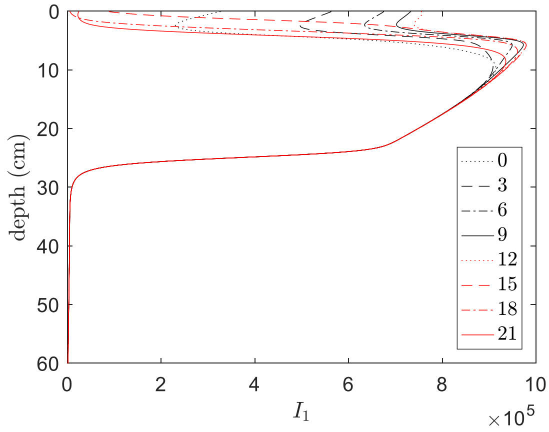

(c) $D_b = 10^{-5}$ and $D_P = 10^{-7}$

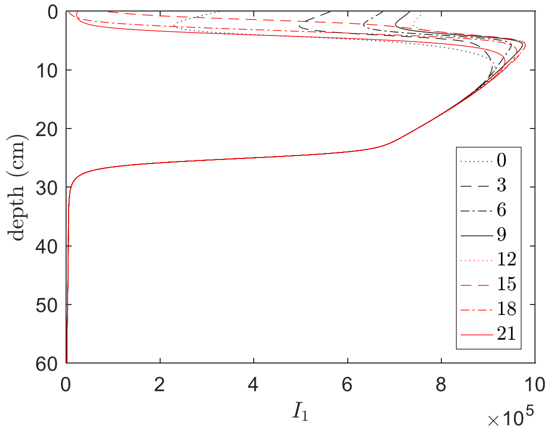

(d) $D_b = 10^{-3}$ and $D_P = 10^{-5}$

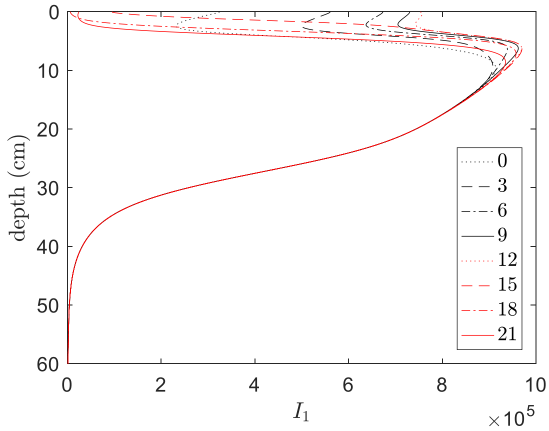

(e) $D_b = 10^{-1}$ and $D_P = 10^{-3}$

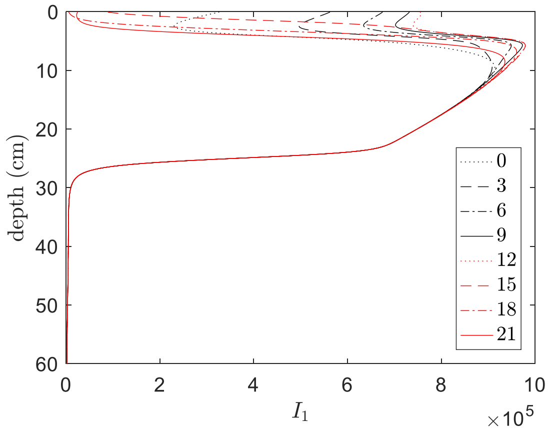

(f) $D_b = 10^{-7}$ and $D_P = 10^{-2}$

Figure 17S. Vertical distributions of infected bacteria in lysogenic stage I_1 in the soil throughout the day on July 1st (Nakhon Phanom province) for different D_p and D_b measured in cm^2/day. Time of the day is indicated in the box of each figure. Model parameters are taken from Table 1 as default values. The unit of the density of I_1 is cell/ml.

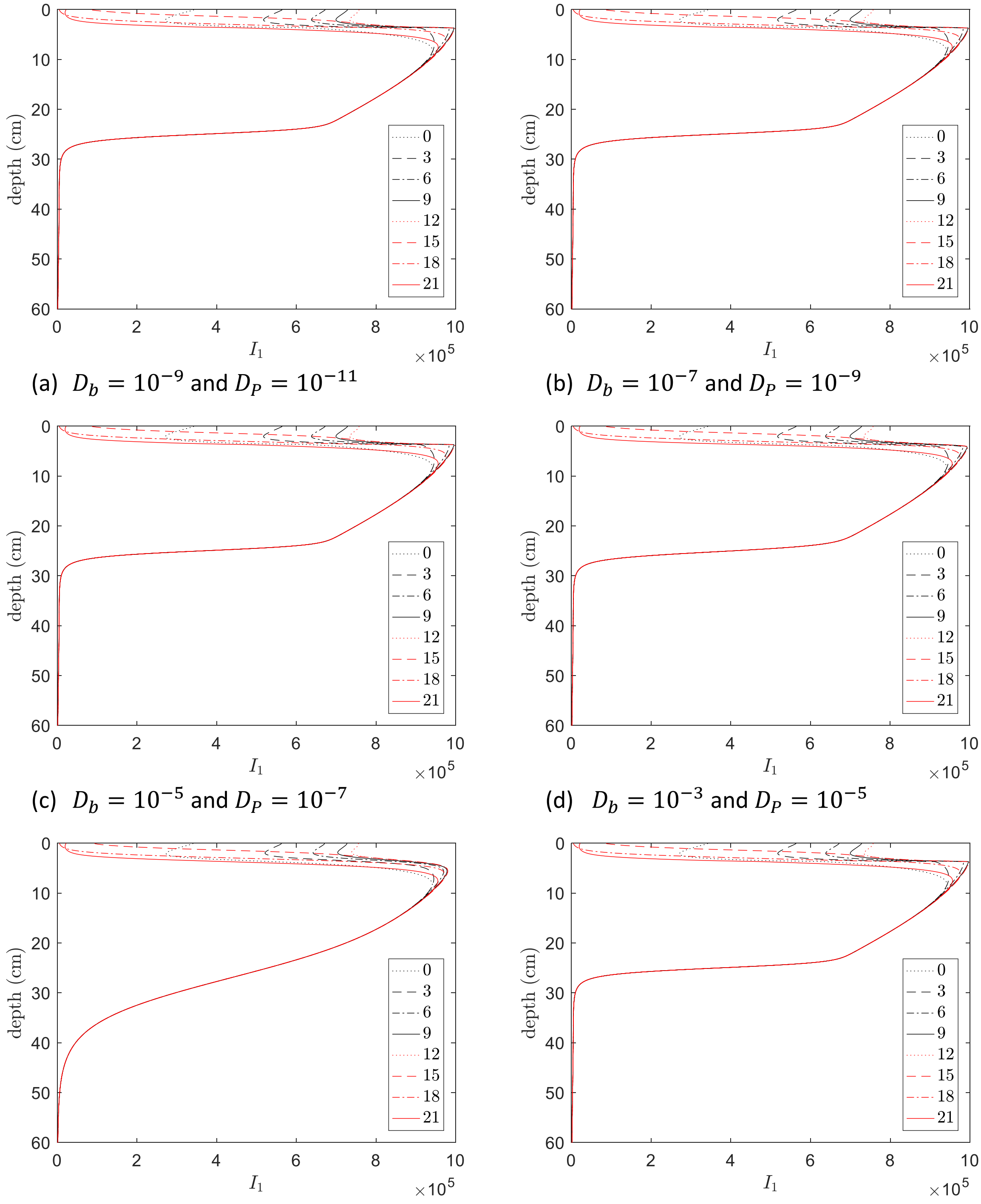

(a) $D_b = 10^{-9}$ and $D_P = 10^{-11}$

(b) $D_b = 10^{-7}$ and $D_P = 10^{-9}$

(c) $D_b = 10^{-5}$ and $D_P = 10^{-7}$

(d) $D_b = 10^{-3}$ and $D_P = 10^{-5}$

(e) $D_b = 10^{-1}$ and $D_P = 10^{-3}$

(f) $D_b = 10^{-7}$ and $D_P = 10^{-2}$

Figure 18S. Vertical distributions of infected bacteria in lysogenic stage I_1 in the soil throughout the day on October 1st (Nakhon Phanom province) for different D_p and D_b measured in cm^2/day. Time of the day is indicated in the box of each figure. Model parameters are taken from Table 1 as default values. The unit of the density of I_1 is cell/ml.

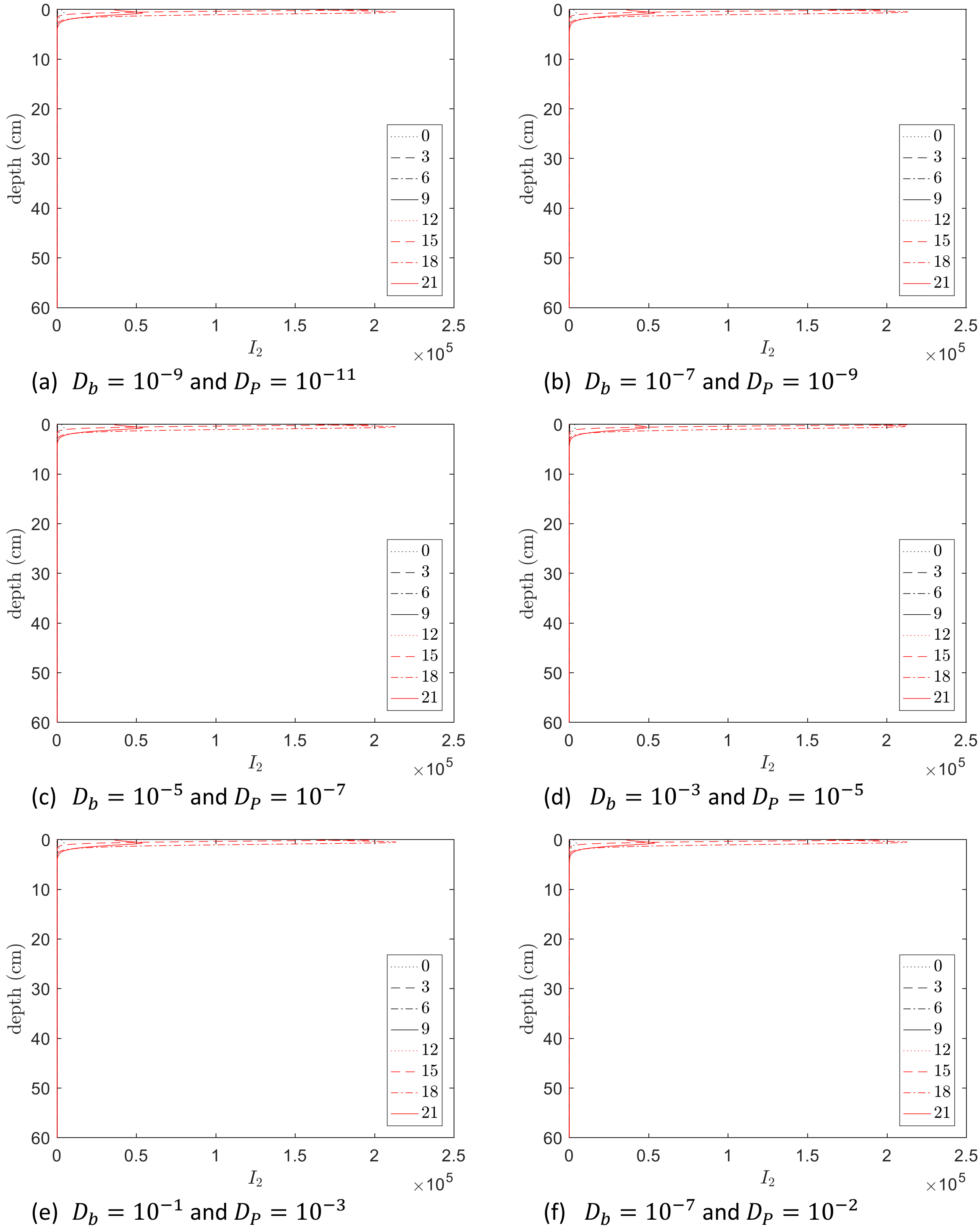

Figure 19S. Vertical distributions of infected bacteria in lytic stage I_2 in the soil throughout the day on January 1st (Nakhon Phanom province) for different D_p and D_b measured in cm^2/day. Time of the day is indicated in the box of each figure. Model parameters are taken from Table 1 as default values. The unit of the density of I_2 is cell/ml.

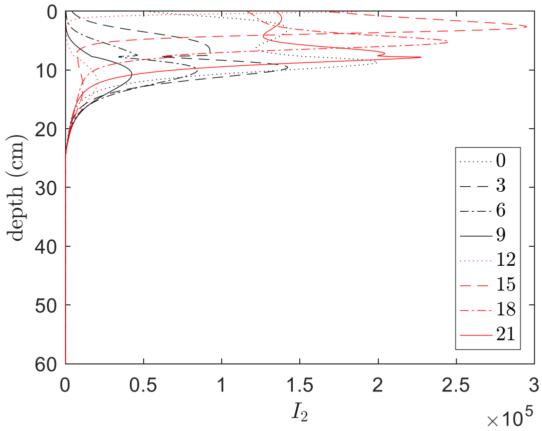

(a) $D_b = 10^{-9}$ and $D_P = 10^{-11}$

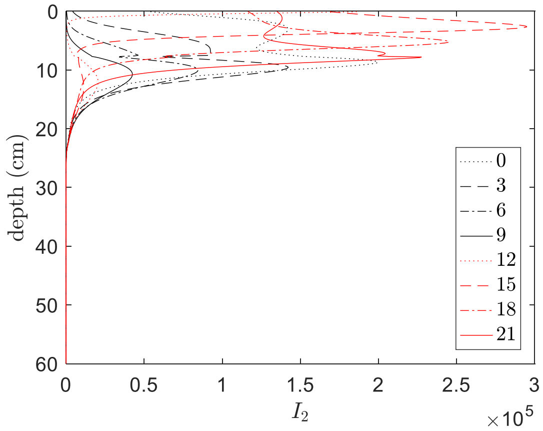

(b) $D_b = 10^{-7}$ and $D_P = 10^{-9}$

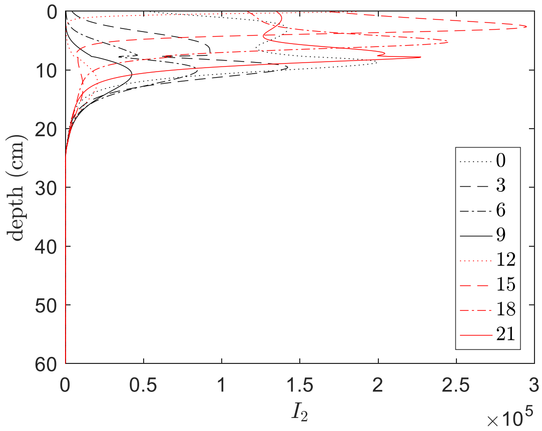

(c) $D_b = 10^{-5}$ and $D_P = 10^{-7}$

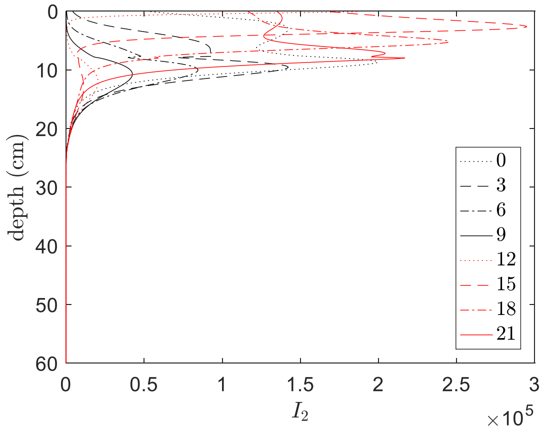

(d) $D_b = 10^{-3}$ and $D_P = 10^{-5}$

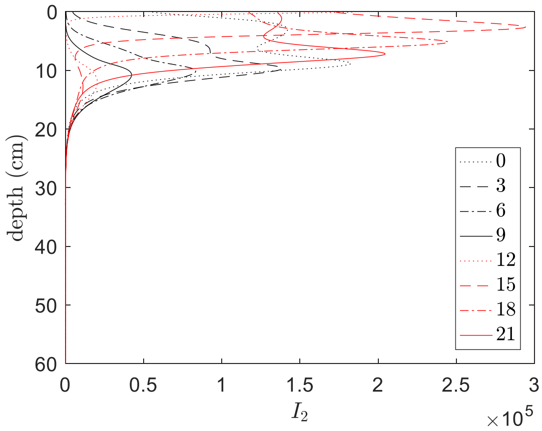

(e) $D_b = 10^{-1}$ and $D_P = 10^{-3}$

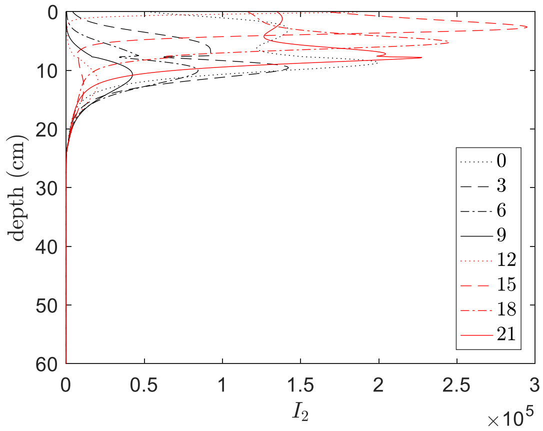

(f) $D_b = 10^{-7}$ and $D_P = 10^{-2}$

Figure 20S. Vertical distributions of infected bacteria in lytic stage I_2 in the soil throughout the day on April 1st (Nakhon Phanom province) for different D_p and D_b measured in cm^2/day. Time of the day is indicated in the box of each figure. Model parameters are taken from Table 1 as default values. The unit of the density of I_2 is cell/ml.

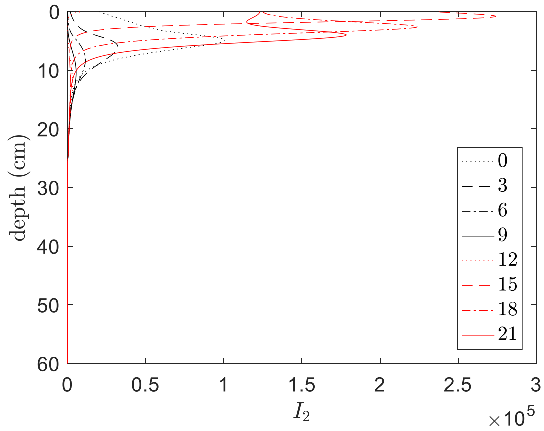

(a) $D_b = 10^{-9}$ and $D_P = 10^{-11}$

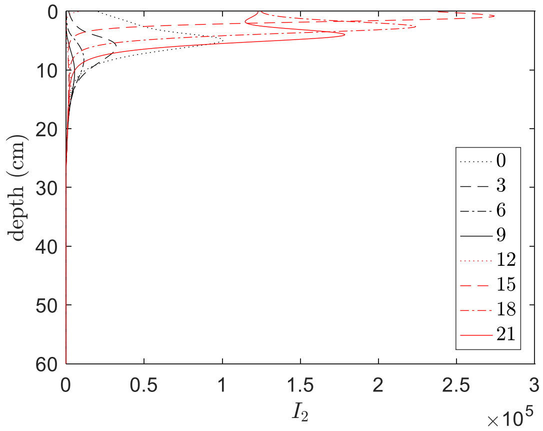

(b) $D_b = 10^{-7}$ and $D_P = 10^{-9}$

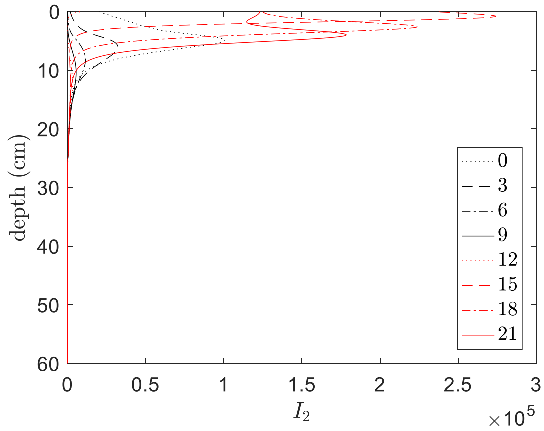

(c) $D_b = 10^{-5}$ and $D_P = 10^{-7}$

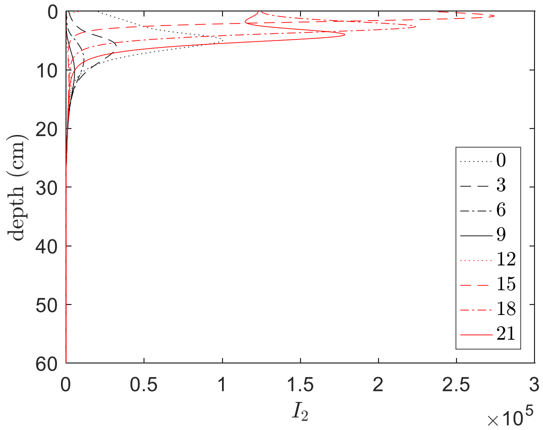

(d) $D_b = 10^{-3}$ and $D_P = 10^{-5}$

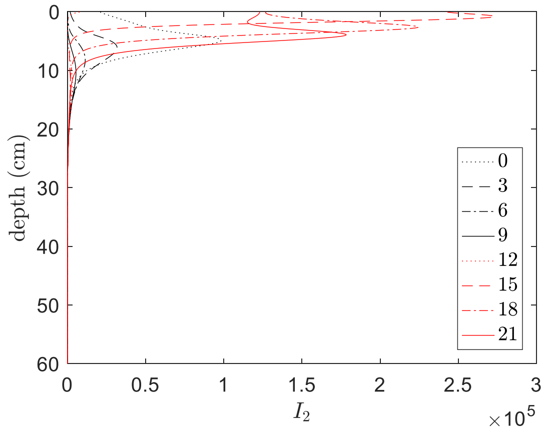

(e) $D_b = 10^{-1}$ and $D_P = 10^{-3}$

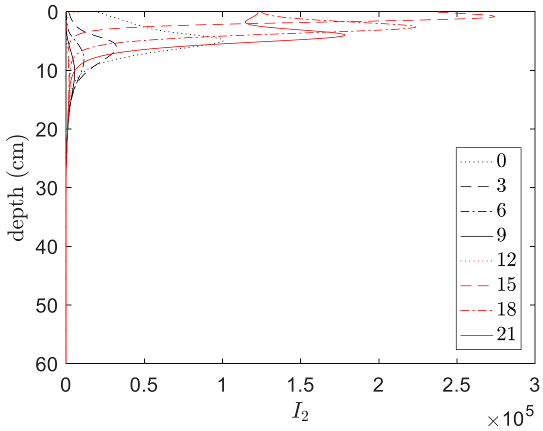

(f) $D_b = 10^{-7}$ and $D_P = 10^{-2}$

Figure 21S. Vertical distributions of infected bacteria in lytic stage I_2 in the soil throughout the day on July 1st (Nakhon Phanom province) for different D_p and D_b measured in cm^2/day. Time of the day is indicated in the box of each figure. Model parameters are taken from Table 1 as default values. The unit of the density of I_2 is cell/ml.

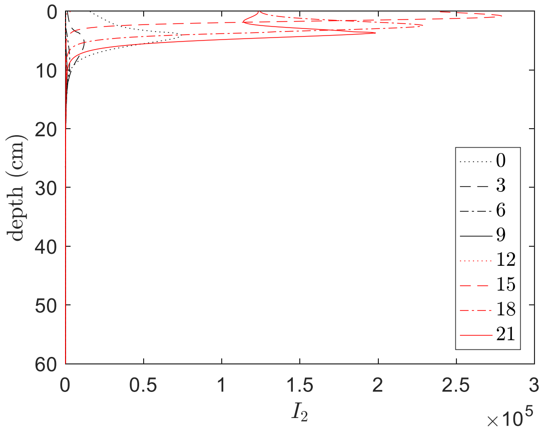

(a) $D_b = 10^{-9}$ and $D_P = 10^{-11}$

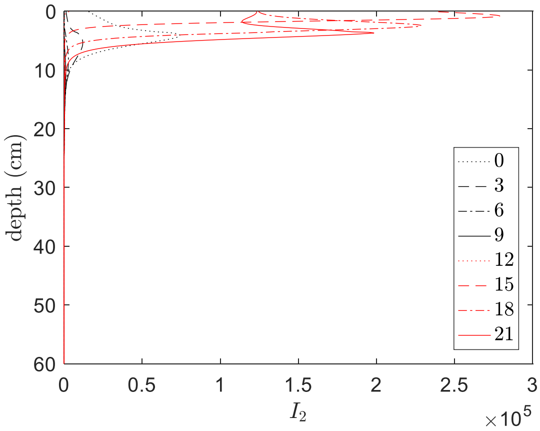

(b) $D_b = 10^{-7}$ and $D_P = 10^{-9}$

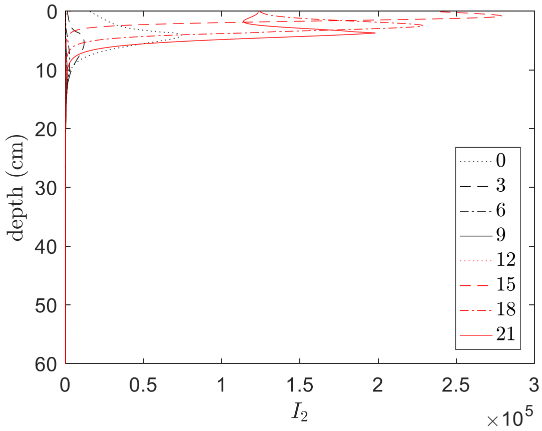

(c) $D_b = 10^{-5}$ and $D_P = 10^{-7}$

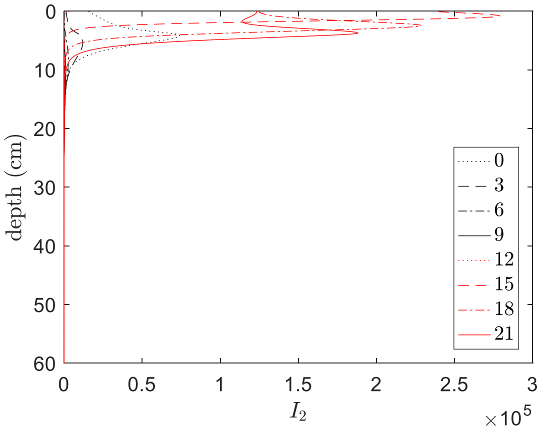

(d) $D_b = 10^{-3}$ and $D_P = 10^{-5}$

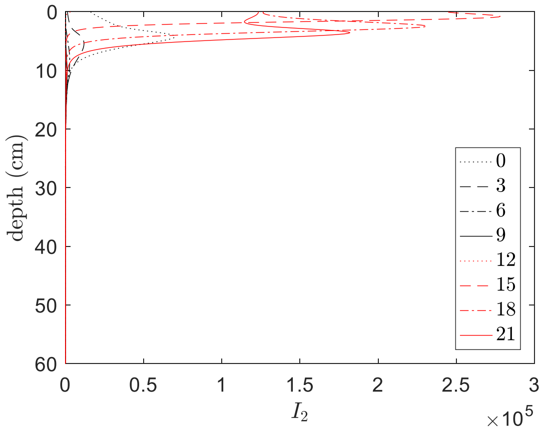

(e) $D_b = 10^{-1}$ and $D_P = 10^{-3}$

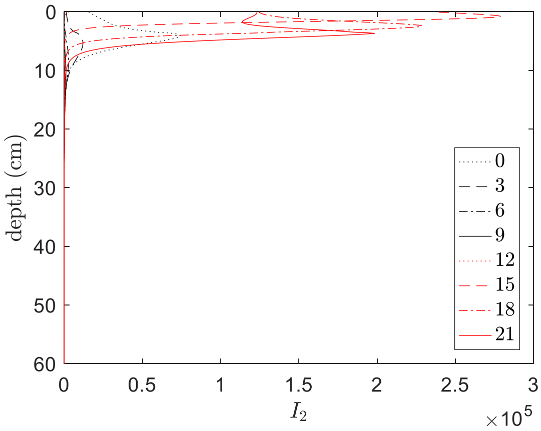

(f) $D_b = 10^{-7}$ and $D_P = 10^{-2}$

Figure 22S. Vertical distributions of infected bacteria in lytic stage I_2 in the soil throughout the day on October 1st (Nakhon Phanom province) for different D_p and D_b measured in cm^2/day. Time of the day is indicated in the box of each figure. Model parameters are taken from Table 1 as default values. The unit of the density of I_2 is cell/ml.

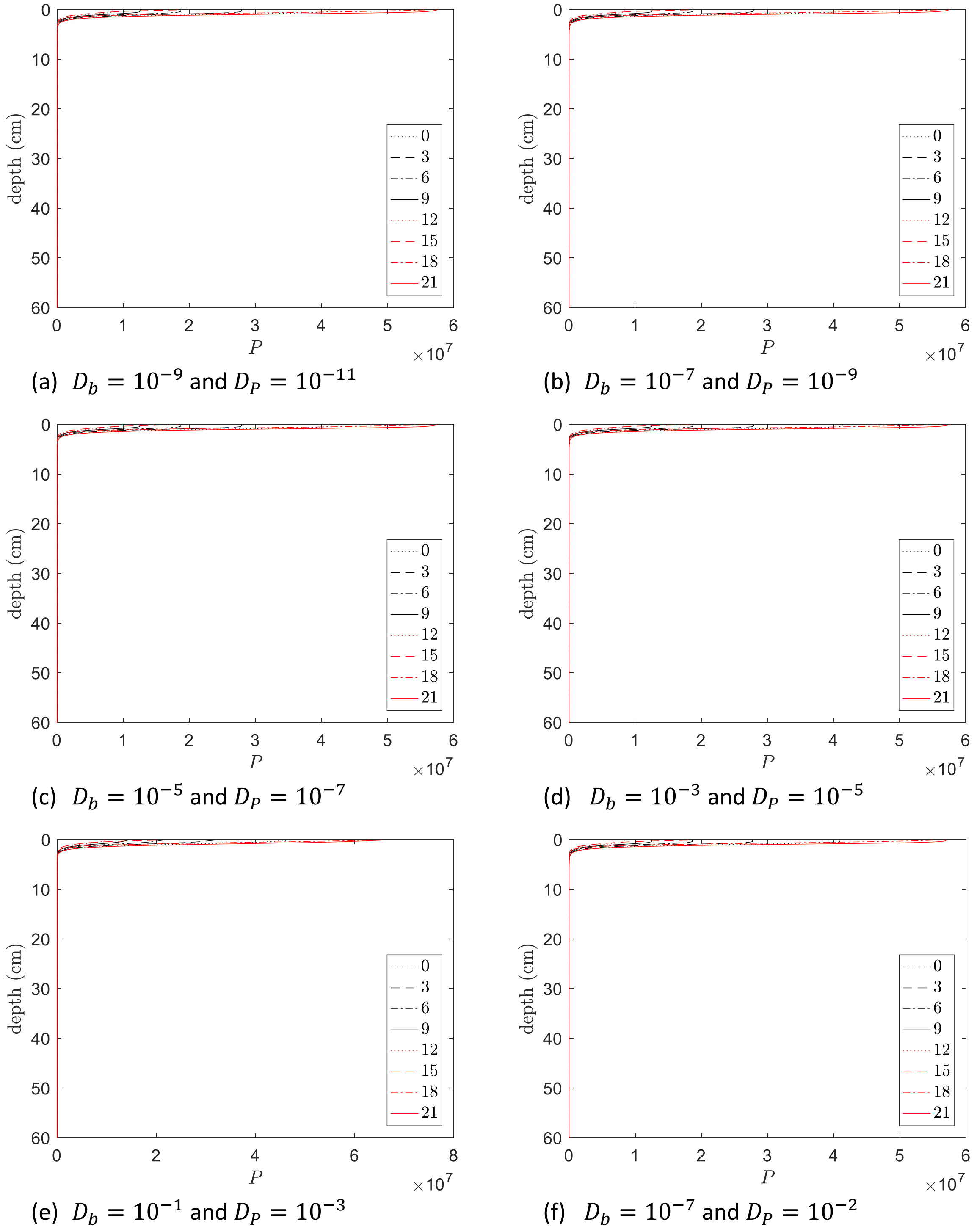

Figure 23S. Vertical distributions of free phages P in the soil throughout the day on January 1st (Nakhon Phanom province) for different D_p and D_b measured in cm^2/day. Time of the day is indicated in the box of each figure. Model parameters are taken from Table 1 as default values. The unit of the density of P is phage/ml.

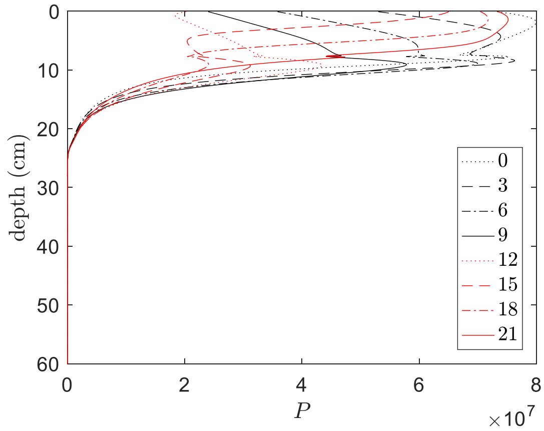

(a) $D_b = 10^{-9}$ and $D_P = 10^{-11}$

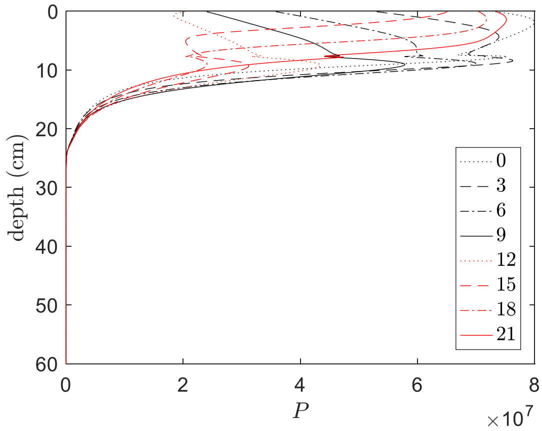

(b) $D_b = 10^{-7}$ and $D_P = 10^{-9}$

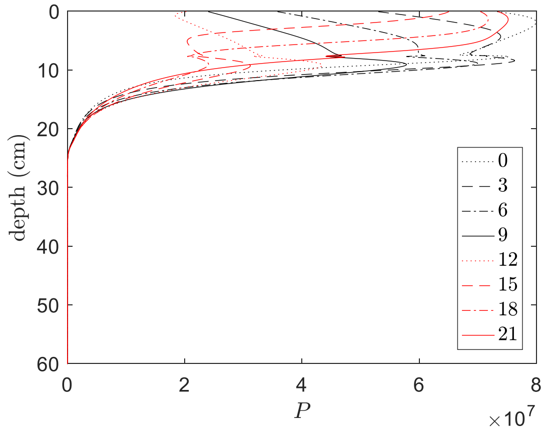

(c) $D_b = 10^{-5}$ and $D_P = 10^{-7}$

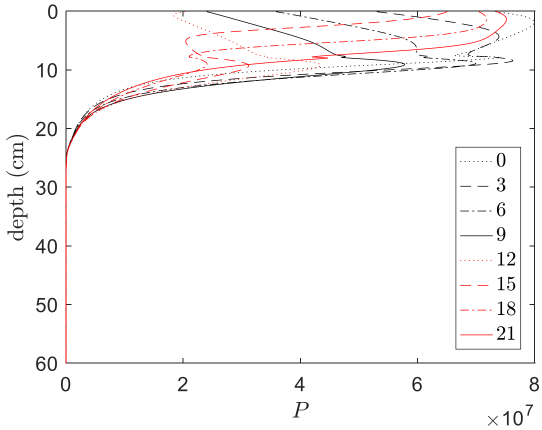

(d) $D_b = 10^{-3}$ and $D_P = 10^{-5}$

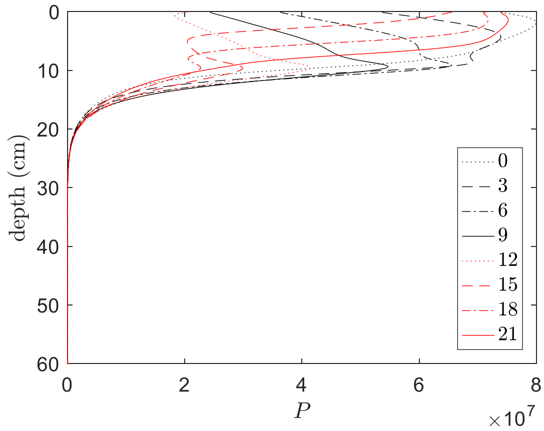

(e) $D_b = 10^{-1}$ and $D_P = 10^{-3}$

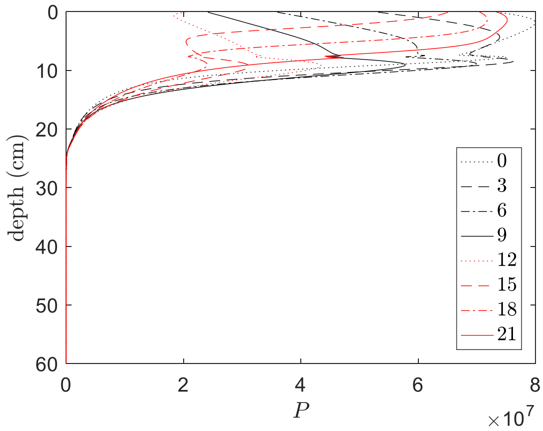

(f) $D_b = 10^{-7}$ and $D_P = 10^{-2}$

Figure 24S. Vertical distributions of free phages P in the soil throughout the day on April 1st (Nakhon Phanom province) for different D_p and D_b measured in cm^2/day. Time of the day is indicated in the box of each figure. Model parameters are taken from Table 1 as default values. The unit of the density of P is phage/ml.

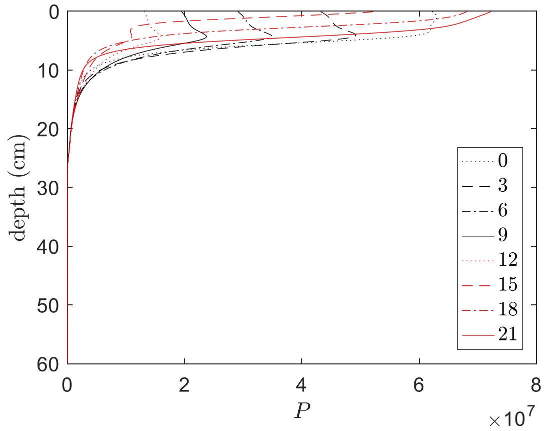

(a) $D_b = 10^{-9}$ and $D_P = 10^{-11}$

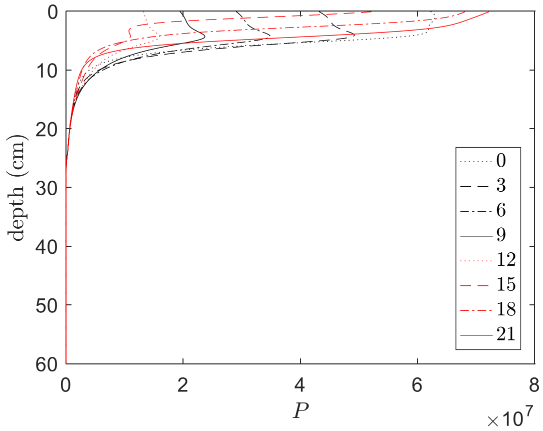

(b) $D_b = 10^{-7}$ and $D_P = 10^{-9}$

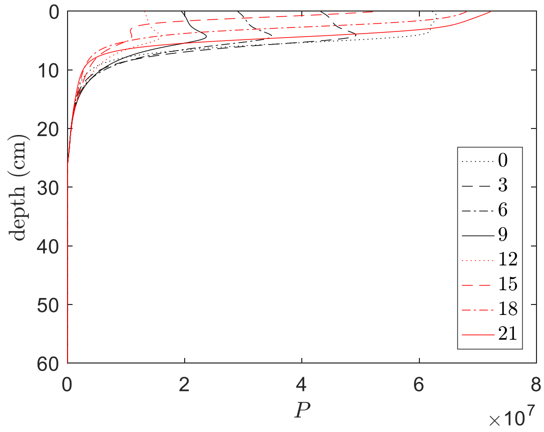

(c) $D_b = 10^{-5}$ and $D_P = 10^{-7}$

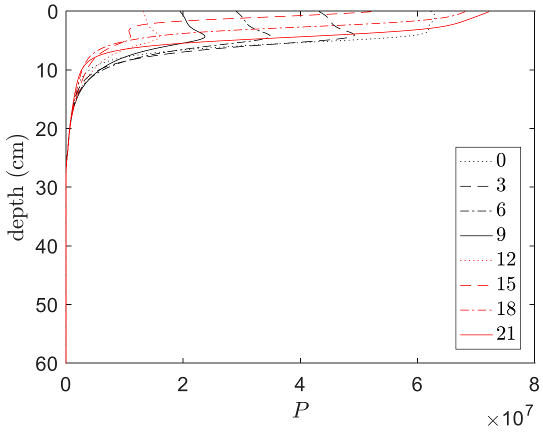

(d) $D_b = 10^{-3}$ and $D_P = 10^{-5}$

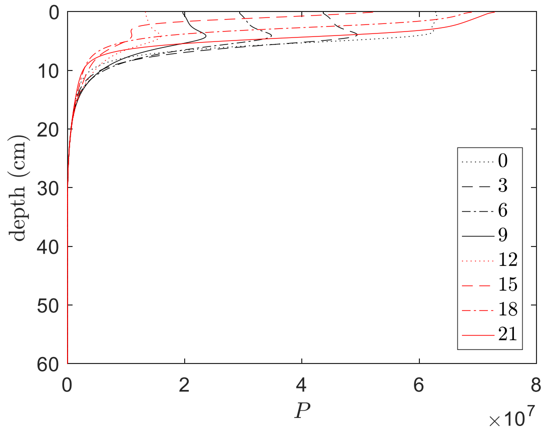

(e) $D_b = 10^{-1}$ and $D_P = 10^{-3}$

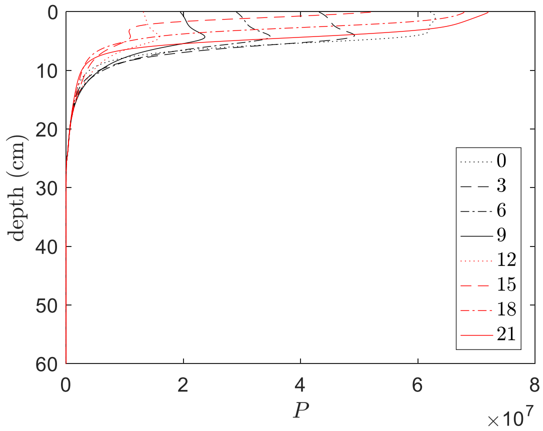

(f) $D_b = 10^{-7}$ and $D_P = 10^{-2}$

Figure 25S. Vertical distributions of free phages P in the soil throughout the day on July1st (Nakhon Phanom province) for different D_p and D_b measured in cm^2/day. Time of the day is indicated in the box of each figure. Model parameters are taken from Table 1 as default values. The unit of the density of P is phage/ml.

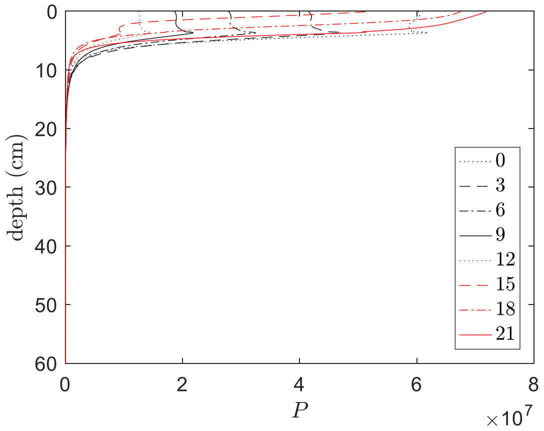

(a) $D_b = 10^{-9}$ and $D_P = 10^{-11}$

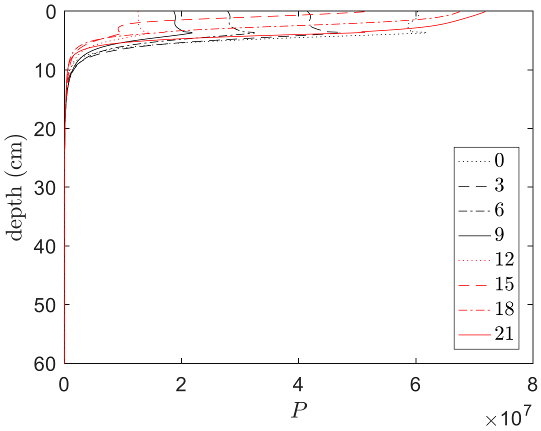

(b) $D_b = 10^{-7}$ and $D_P = 10^{-9}$

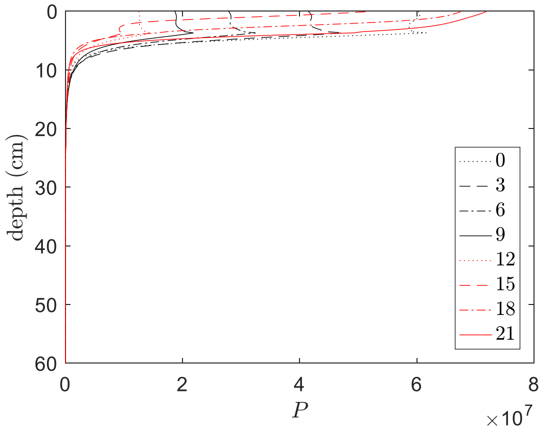

(c) $D_b = 10^{-5}$ and $D_P = 10^{-7}$

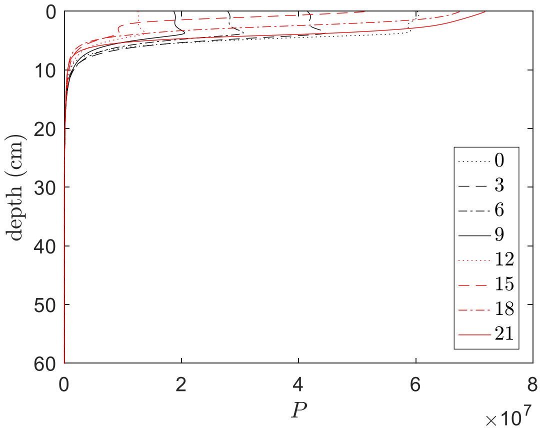

(d) $D_b = 10^{-3}$ and $D_P = 10^{-5}$

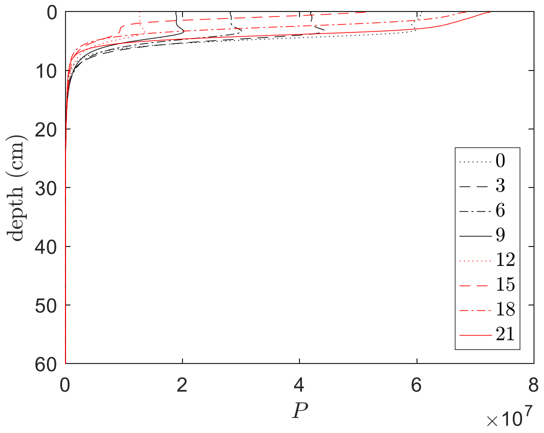

(e) $D_b = 10^{-1}$ and $D_P = 10^{-3}$

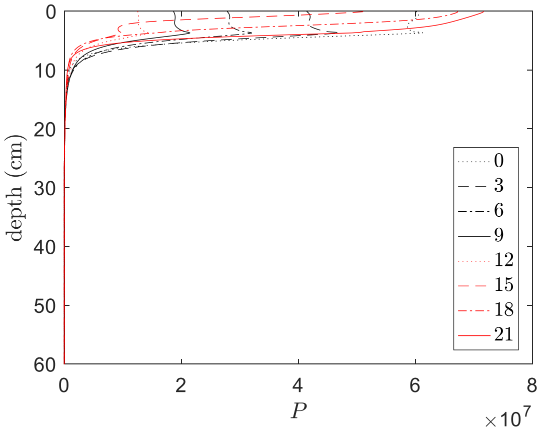

(f) $D_b = 10^{-7}$ and $D_P = 10^{-2}$

Figure 26S. Vertical distributions of free phages P in the soil throughout the day on October1st (Nakhon Phanom province) for different D_p and D_b measured in cm^2/day. Time of the day is indicated in the box of each figure. Model parameters are taken from Table 1 as default values. The unit of the density of P is phage/ml.

SM6

In Figures 27S-28S we show the spatiotemporal dynamics of bacteria-phage interaction predicted by the model for Sa Kaeo province in Thailand. The model parameters are taken as default values from Table 1. The considered temperature time series correspond to the three year period 2013-2016. They are obtained from the website www.worldweatheronline.com (accessed 11 April 2017). They are also presented in Figure 3 in the paper by Egilmez et al 2018 (Scientific Reports, 8:9642)

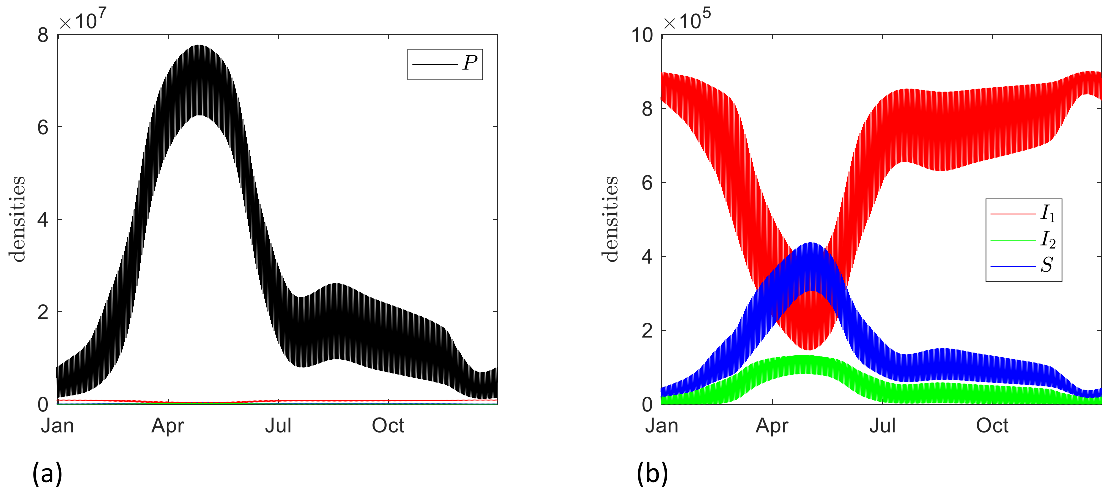

Figure 27S: (a), (b) Daily and seasonal temporal dynamics of bacteria and phage numbers within the upper 20 cm of the soil predicted by the model calculated for Sa Kaeo province in Thailand. Model parameters are taken from Table 1 as default values. The unit of the densities of bacteria and phages are cell/ml and phage/ml, respectively.

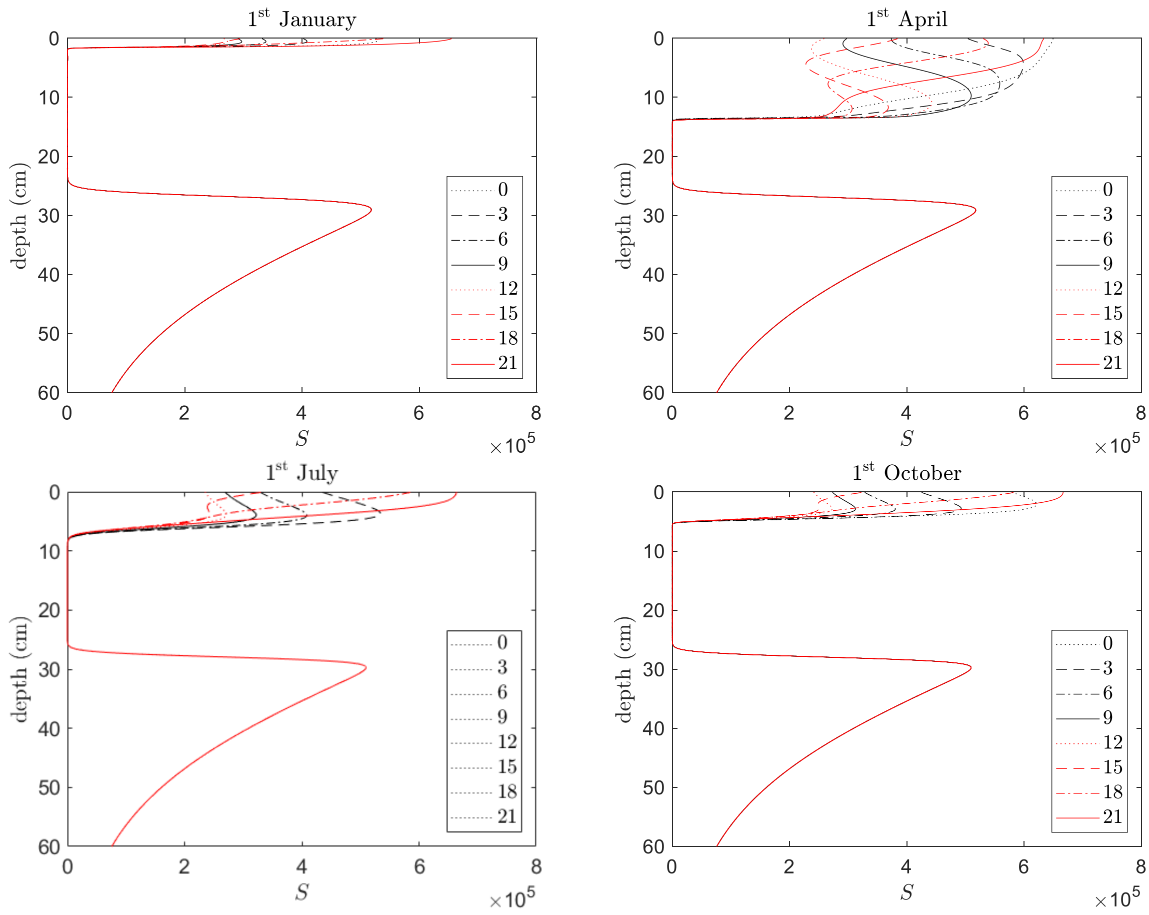

Figure 28S. Vertical distributions of susceptible bacteria *S* in the soil throughout the day and the year predicted by the model calculated for Sa Kaeo province. Time of the day is indicated in the box of each figure. Model parameters are taken from Table 1 as default values. The unit of the density of *S* is cell/ml.
